# A 15 Year Evaluation of West Nile Virus in Wisconsin: Effects on Wildlife and Human Health

**DOI:** 10.3390/ijerph17051767

**Published:** 2020-03-09

**Authors:** Johnny A. Uelmen, Charles Brokopp, Jonathan Patz

**Affiliations:** 1 Department of Population Health Sciences, University of Wisconsin, 610 Walnut Street, 707 WARF Building, Madison, WI 53726, USA; cbrokopp@gmail.com (C.B.); patz@wisc.edu (J.P.); 2 Department of Pathobiology, University of Illinois, 2001 South Lincoln Avenue, Urbana, IL 61802, USA; 3 Wisconsin State Laboratory of Hygiene, 2601 Agriculture Drive, P.O. Box 7904, Madison, WI 53718, USA; 4 Nelson Institute for Environmental Sciences, University of Wisconsin, 258 Enzyme Institute, 1710 University Avenue, Madison, WI 53726, USA

**Keywords:** West Nile virus, disease ecology, public health entomology, mosquito-borne disease, GIS

## Abstract

West Nile virus (WNV) is the most important and widespread mosquito-borne virus in the United States (U.S.). WNV has the ability to spread rapidly and effectively, infecting more than 320 bird and mammalian species. An examination of environmental conditions and the health of keystone species may help predict the susceptibility of various habitats to WNV and reveal key risk factors, annual trends, and vulnerable regions. Since 2002, WNV outbreaks in Wisconsin varied by species, place, and time, significantly affected by unique climatic, environmental, and geographical factors. During a 15 year period, WNV was detected in 71 of 72 counties, resulting in 239 human and 1397 wildlife cases. Controlling for population and sampling efforts in Wisconsin, rates of WNV are highest in the western and northwestern rural regions of the state. WNV incidence rates were highest in counties with low human population densities, predominantly wetland, and at elevations greater than 1000 feet. Resources for surveillance, prevention, and detection of WNV were lowest in rural counties, likely resulting in underestimation of cases. Overall, increasing mean temperature and decreasing precipitation showed positive influence on WNV transmission in Wisconsin. This study incorporates the first statewide assessment of WNV in Wisconsin.

## 1. Introduction

Since its introduction in 1999, the West Nile virus (WNV; Family Flaviviridae) has become the most important mosquito-borne disease in the United States (U.S.) and one of the most widespread in the world [1,2,3,4]. WNV is a member of the Japanese encephalitis virus serogroup, a collection of viruses that cause human encephalopathy [5]. WNV is primarily amplified by infecting avian hosts, but will also infect humans, horses, and other mammals. Infected mammalian hosts are considered “dead-end” hosts because they do not produce high enough viremia to infect subsequent feeding mosquitoes, effectively ending the transmission cycle. While clinical manifestation can be severe in humans, approximately 80% of all infected humans are asymptomatic—20% develop West Nile fever and among these, approximately 1% will develop severe, and sometimes deadly, neuroinvasive disease [6,7].

WNV engages in an enzootic cycle, requiring both mosquitoes and birds to complete its transmission cycle. Mosquitoes from the *Culex* (*Cx.*) genus are the primary vectors for transmitting WNV. At least 160 bird species (Petersen et al. 2013 reports that WNV has been detected in 326 bird species in the United States, but not all are capable of amplifying virus to infect subsequent biting mosquitoes), particularly those from the largest order, *Passerines,* are known to amplify WNV in the U.S. [8]. Birds from the family Corvidae (e.g., crows, ravens, jays) are highly susceptible, early-onset hosts and are classified as key sentinel species for WNV surveillance, serving as a reliable proxy for indicating disease prevalence in an ecosystem [9,10].

Seasonal and microclimatic temperature, precipitation trends, and occurrence of extreme climatic conditions influence *Culex* biology and behavior, flight activity, viral replication rates, and mosquito reproduction, thus affecting WNV transmission [11,12,13]. Outbreaks of other arboviral encephalitides, including Saint Louis encephalitis virus (SLEV), have been recorded in the Middle East, in Eastern Europe, New York, and in California, all following droughts and/or periods of record high temperatures [14,15]. However, other mosquito-borne diseases, such as dengue and malaria, thrive during rainy seasons and flooding events [16,17,18,19]. 

Despite education campaigns and efforts to control for mosquito populations, WNV continues its epizootic and zoonotic cycle year to year, with large-scale outbreaks occurring intermittently across the country [20]. Accurately predicting when and where WNV transmission will occur, especially prior to outbreak years, has been difficult to assess. Detailed WNV assessments have rarely occurred across statewide levels and have predominantly occurred in urban regions, where disease cases are most concentrated [21,22].

The state of Wisconsin, in close proximity to Illinois, a state known for high annual WNV activity, does not have a detailed study of statewide epidemiology, nor publicly funded active mosquito control programs (e.g., mosquito abatement districts) to actively monitor disease-related trends (mosquito infection rates, disease prevalence in captured avian hosts, etc.) [23]. Wisconsin has engaged in active surveillance of WNV, but statewide efforts were limited to the first six years after introduction of disease (2001) and mainly targeted local jurisdictions. Since 2007, the state has relied on passive surveillance, a method that requires few resources and identifies disease trends over time. However, the degree of compliance and timeliness in passive reporting varies widely. The reliance upon passive surveillance, high variability in annual disease incidence, and a large number of asymptomatic cases may have led to large underestimations of true disease prevalence.

While many host–pathogen relationships have been studied and modeled in the past, high-resolution spatiotemporal models are needed to address region- and virus-specific effects on the health of human, animals, and the environment [24], an approach coined One Health [25]. The goal of this paper is to use all known available wildlife, human, and environmental data to develop a statewide disease model that will provide a foundation for understanding the ecology and prevalence of WNV in the state of Wisconsin. 

## 2. Methods

### 2.1. Study Location

The state of Wisconsin is located in north-central U.S., a region classified as a temperate climate zone. Wisconsin is the 23rd largest (169,640 km^2^) and 20th most populous (5,778,708) state in the U.S. [26].

### 2.2. WNV Case Data

Data from the time period of 1 January 2001 to 28 February 2016 was retrospectively collected from various federal and state agencies. Rates of disease refer to the number of cases for a specific species group divided by the total number of species submitted. Incidence of disease refers to the number of human cases divided by the total population. All rates and incidence are analyzed at the county level.

The United States Geological Survey (USGS) National Wildlife Health Center (NWHC) provided a total of 1178 records of avian and non-human mammal species affected by WNV. Data queries from USGS were filtered in SAS 9.4 (SAS Institute Inc., Cary, NC, USA) [27] to include only Wisconsin and have any combination of the following keywords: West Nile, encephalitis, seropositive, WNV, avian species and West Nile fever. At the time of this project’s analysis, USGS NWHC had not yet finalized a final case definition for WNV in wildlife. All records were individually reviewed, reviewing field notes from the attending veterinarians and/or wildlife biologists (if available) as well as clinical and laboratory findings. The Wisconsin Division of Health Services (DHS) provided a total of 15,690 avian and non-human mammal records and all 239 human cases (confirmed = 230, probable = 9). Suspected human cases were not available at the time of analysis. To ensure privacy, human and wildlife data was provided at a county aggregate level. No personal information, address, or geo-location can be identified for any submitted case, resulting models, tables, or figures in any form. 

The Wisconsin Department of Natural Resources (DNR) provided a total of 2024 records of avian and other wildlife species affected by WNV. The Wisconsin Veterinary Diagnostic Laboratory (WVDL) provided all 1561 WNV equine records. 

To reduce the possibility of duplicate records that may have been submitted to two or more institutions, each submission was cross-validated by the unique identification number. If a unique identification number was not available, the authors stratified submissions by species, collection date, and location, and eliminated any duplicates. 

### 2.3. Final Case Definition

Each submission’s laboratory criteria was reviewed (by order of priority) for: 1. Viral detection (and/or isolation) in tissue, blood, CSF, or other body fluid, 2. Compatible clinical symptoms (humans) or signs presented (wildlife) (alive in field or captivity or lesions observed during necropsy), or 3. Virus-specific IgM antibodies ≥ 1:40 dilution). To reliably indicate active infection, a threshold of IgG antibody remaining in the body (titer) has been established as ≥ 1:40 dilution [28].

Confirmed WNV cases were determined if isolation and/or PCR detection of the virus occurred and compatible clinical symptoms were presented. Probable WNV cases were determined if virus isolation and/or detection occurred, but no compatible clinical signs were observed or reported. Cases were also determined as WNV probable if compatible clinical signs were observed with virus-specific IgM antibodies present. Suspected cases displayed WNV-specific (IgM) antibodies at an antibody titer level >1:40 dilution or compatible clinical signs were present. Exposure was determined as any WNV-specific (IgM or IgG) antibody level with no presentation of compatible clinical signs. A WNV-positive case is determined as any species submitted for testing and determined to be positive or probable. Each agency’s final case definitions are located in Table 1.

### 2.4. Climate Data

All temperature and precipitation data was acquired from the Midwestern Regional Climate Center’s (MRCC) application tools environment [29]. Mean and maximum daily values were acquired for temperature (°F) while daily mean, daily maximum, and total monthly data was acquired for precipitation (in.). A total of 541 reporting stations from each of Wisconsin’s 72 counties, except Menominee County, provided climate data. The values for Menominee County (no stations reporting) were averaged from its neighboring county values.

### 2.5. Elevation and Land Cover

Land cover and corresponding elevational changes were included to capture the wide diversity of potential habitats and ecological niches associated with the biology of mosquito vectors [30,31]. The University of Wisconsin—Green Bay’s Wisconsin Bedrock Elevation Map was used to create Wisconsin’s land elevation data [32]. Land cover was acquired by georeferencing the Wisland-2 map [33], assessing forest, urban, grassland, agricultural, shrubland, wetland, and water as a percentage in each county (Table S1).

### 2.6. Statistical Methods

Using logistic regression (dependent variable: presence/absence of a WNV case) and linear regression (dependent variable: annual rate of disease), the combined effects of the following explanatory variables were assessed by month and year for each Wisconsin county (n = 72): climatic (temperature: monthly mean and maximum; precipitation: daily mean, daily maximum, and monthly total), environmental (land cover: % of county agriculture, forest, grassland, shrubland, urban, water, and wetland), average county elevation, and county population, on WNV prevalence for all avian species, equines, and humans. Corvids, as sentinel species that constitute the majority of all avian species submitted, were categorized independently for this study. Multivariate multiple regression models were assessed using the generalized linear model (GLM) and logistic regression procedures (PROC GLM and PROC LOGISTIC, respectively), in SAS 9.4 [27]. Regression values, calculated using the PROC REG procedure in SAS 9.4, compose the ecological modeling for WNV and inform the relative effects (positive or negatively associated with disease) and magnitude (weighted values of disease effect) of specific environmental or climatic factors for each specimen group (avian, corvid, equine, or human) submitted for testing. The selection of final explanatory variables was based on overall lowest Akaike information criterion (AIC) values via backward elimination, as this method reduces both model over-fitting and collinearity between variables [35,36]. Data was organized, processed, and visualized using JMP 11.0.0 (SAS Institute, Cary, NC, USA) [27]. GLM results are reported (type III error).

### 2.7. Geospatial Analyses

To evaluate any spatial dispersal or clustering relationships associated with WNV cases, spatial autocorrelation and hot spot analyses were conducted, indicated by the test statistics, Moran’s I (−1 = dispersed, +1 = clustered) and Getis-Ord Gi* (hot = red, cold = blue), respectively. Both the autocorrelation and hot spot analysis were conducted using the spatial statistics package in ArcGIS 10.5 [37]. Results from the linear and logistic regression model were interpolated using a kriging analysis (Empirical Bayesian method). Empirical Bayesian kriging was chosen for the prediction analysis as it contains a distribution of semivariograms and is known to be an accurate assessment for Gaussian distributed datasets [38]. Kriging analyses were conducted using the interpolation toolset in the ArcGIS 10.5 geostatistical analyst package. For all statistical analyses, significance was determined by corresponding *P* values of <0.05 as ‘significant’ and 0.05 < *P* < 0.10 as ‘marginally significant’.

## 3. Results

In Wisconsin from 2001 to 2016, 20,691 specimens were submitted for WNV testing (avian = 18,444, 89.1%, mammalian = 2247, 10.9%), representing 63 families (Table 1). Overall, 50 species from 26 families were confirmed positive for WNV (Table 2). The majority of avian cases were from the family Corvidae, particularly among the American crow. WNV submissions peaked between 2002 and 2008 for all species, accounting for 71.0% of all specimens submitted for testing. 

**Figure 1:**
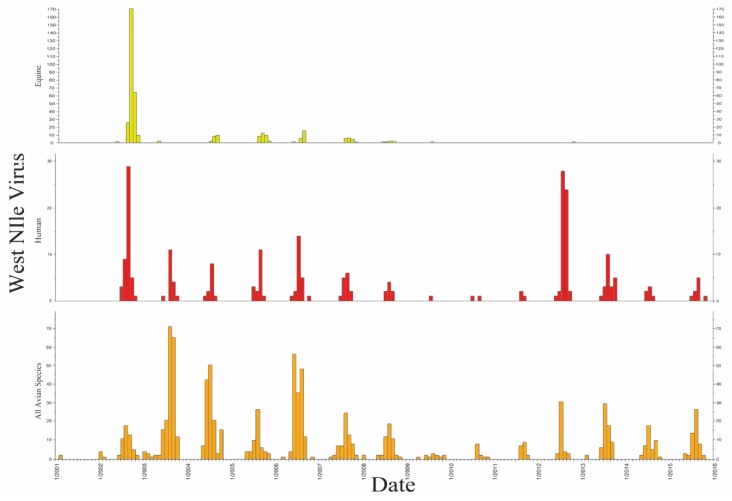
Confirmed Wisconsin WNV cases for equine (**A)**, human (**B)**, and all avian species (**C)** submitted by week, 2001–2016.

There were 239 confirmed human cases, resulting in 17 deaths (7.1%). Human cases were highest in 2002 (n = 46, 19.2%) and in 2012 (n = 56, 23.4%) (Figure 1), with a cumulative incidence of 4.24 per 100,000 people (Table 3). The year 2012 was the deadliest for humans (n = 5), resulting in a higher average annual incidence (1.00 per 100,000 people) than any other year. Equines also experienced a spike in the number of initial cases in early years, peaking in 2002 (n = 270, 17.3%). Since the introduction of the equine WNV vaccine in 2004 [39,40], cases have dramatically lessened and remained at primarily low levels.

The three most populous counties in the state, Milwaukee, Dane, and Waukesha, were the most heavily sampled, totaling 6730 total submissions (all species) (n = 2308, 3187, and 1235, respectively), or 32.5% overall, but totaling only 224 total cases (3.3% case rate vs. 7.7% state case rate). Rural Pepin (n = 7, 30.4%), Calumet (n = 3, 23.1%), and Jackson (n = 11, 21.6%) had the top three highest overall WNV case rates, respectively (Table S2). 

**Figure 2:**
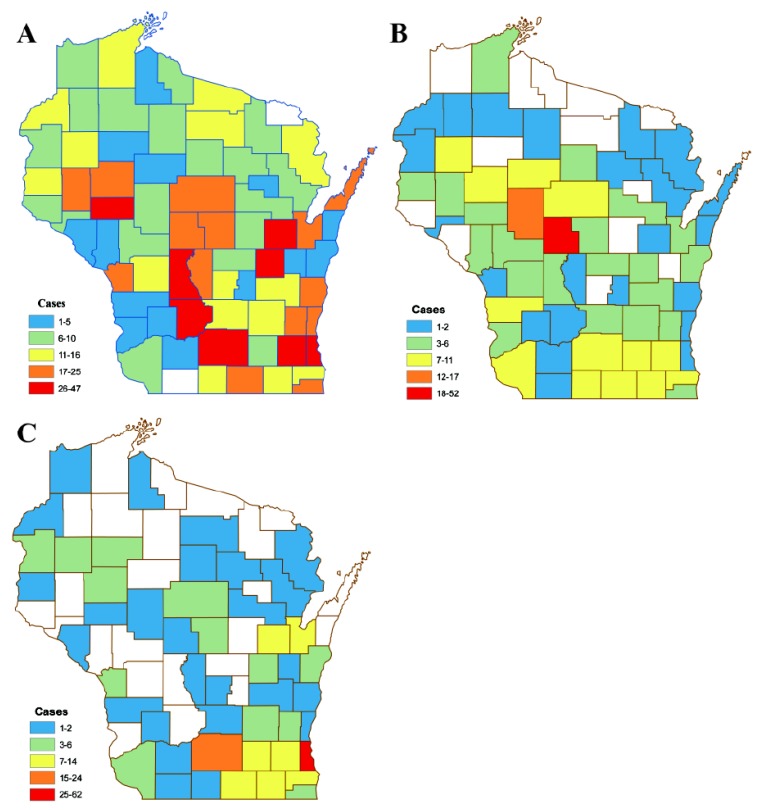
Wisconsin choropleth map of total confirmed positive and probable West Nile virus cases by county for all avian (**A**), equine (**B**), and human (**C**) species, 2001–2016. Values are defined based on quintiles of confirmed and/or probable cases. Counties in white have no positive confirmed WNV cases.

Seventy-one of 72 Wisconsin counties (98.6%) reported at least 1 positive case from any species (Figure 2). Positive avian cases were found in 70 of 72 counties (97.2%) while positive mammalian cases were found in 61 of 72 counties (84.7%). All counties submitted samples for WNV, but total numbers varied significantly (*p* < 0.0001), from a high of 3187 (Dane) to a low of 9 (Florence).

**Figure 3:**
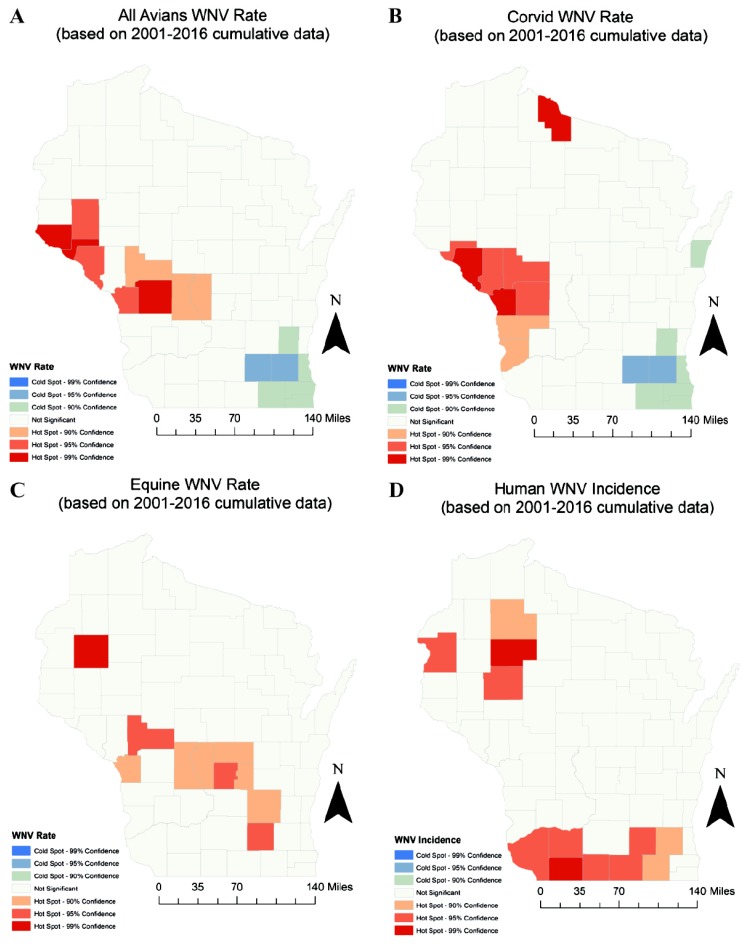
Hot spot analysis for WNV in Wisconsin (all years pooled), by all avian (**A**), corvid (**B**), equine (**C**), and human (**D**) cases. Red areas indicate statistical clustering while blue areas indicated statistical dispersal relationships. Yellow areas indicate non-significance and white areas had no case to assess.

All submitted species were evaluated for any spatial autocorrelation as indicated by Moran’s I and respective *P* values. Species were analyzed for all years (pooled) (Figure 3) and by the WNV “peak years” 2002 and 2012 (Figure S1). The following species and respective year combination reported a significant clustered relationship with WNV: 2002 corvids (*p* = 0.0244), 2012 avians (*p* = 0.0007), 2012 corvids (*p* = 0.009), all years (pooled) avians (*p* = 0.0085), and all years (pooled) corvids (*p* = 0.051) (Table 4). Only 2012 equines (*p* = 0.0032) were found to have a dispersal relationship with WNV. No random relationships were observed.

Regression analyses (for all years, pooled) revealed significantly different effects of climatic and land use variables on WNV rates by species (Table 5). Only the linear regression assessment of wetlands displayed a positive association with WNV rates across all species. Human WNV rates were positively associated with high mean monthly temperatures (°F) and were found predominantly in grassland, urban, or wetland. Low annual precipitation (in.) was the only climatic factor negatively associated with human WNV rates. With the exception of average daily accumulation (*p* = 0.0198), all other temperature and precipitation variables were not statistically significant for statewide whole model estimates (Appendix A Table A1 and Table A2).

**Figure 4:**
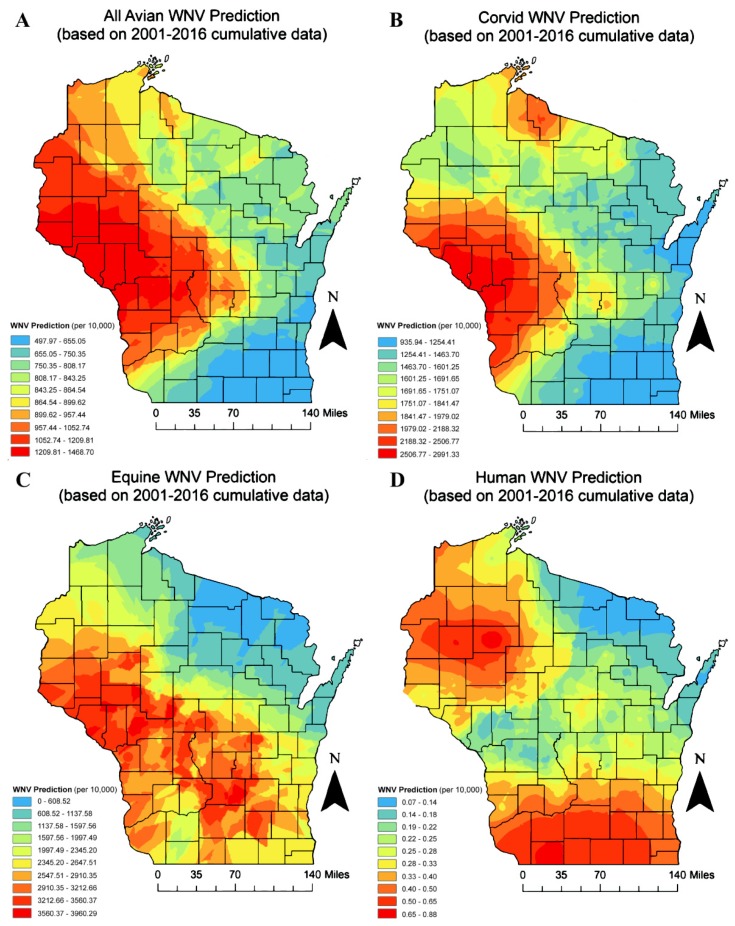
Wisconsin WNV prediction maps by all avian cases (**A**), corvids (**B**), equines (**C**), and humans (**D**) (Empirical Bayesian kriging method) based on cumulative (2001–2016) case data. Color values indicate predicted WNV cases per individual species.

Overall, WNV is predicted to have the highest rates for any species in the northwestern, rural counties of Wisconsin (Figure 4). The highly populated southeast region of the state is predicted to have lower WNV rates for all avian (pooled) and corvid species. Northeastern regions of the state, especially those counties bordering the Upper Peninsula with Michigan, are predicted to have low WNV rates.

## 4. Discussion

Modeling WNV is difficult due to uncertainty and reliability in both the quantity and quality of data. Infected but asymptomatic humans contribute to low case prevalence, disease surveillance and testing efforts are inconsistent, and epidemics are highly variable. WNV surveillance efforts were often allocated to regions with high human populations, resulting in a strong correlation (r^2^ = 0.724) of positive cases with increasing population (Figure S2). While this is expected, rural and less densely populated urban centers are likely underrepresented. In this study, WNV incidence was highest in rural populations, corroborating several other studies (Gates and Boston 2009; Sugumaran et al. 2009; [13,41,42,43]. Counties with the highest human populations have the greatest density of health care resources (e.g., providers, diagnostic laboratories), and this may have resulted in higher frequencies of WNV detection. Due to the seasonality of WNV activity, the greatest exposure potential to humans occurs in mid to late summer months, a period of higher frequency of outdoor activities and travel. Additionally, many human cases were likely exposed outside of the jurisdiction where the final report was generated.

Spatial analyses for epidemiological data are most robust when utilizing point-specific geo-locations. However, to ensure privacy, human data was analyzed at the county aggregate level. When analyzing high case-density locations, like Dane, Milwaukee, and Waukesha, greater ecological inferences can be made using geo-specific point data for each case, rather than aggregating at the entire county level.

Additional research would greatly contribute to the understanding of WNV disease ecology by focusing on the periodicity of immunologically naïve hosts and overwintering mechanisms of the virus, as well as the spatiotemporal trends and meal analyses of known bridge vectors. It is plausible that cases spiking in 2002 and later in 2012 may have resulted from immunologically naïve hosts [44,45]. Studies have suggested that the American robin (*Turdidae*) may play a significant role in the transmission of WNV, finding that a majority of WNV infectious mosquitoes, particularly *Cx. pipiens*, preferred the robin to a variety of other avian species [5,46,47,48,49]. However, Janousek et al. [50] found that communal Robin roosts in urban areas may decrease biting rates, reducing WNV infection prevalence in mosquitoes.

While this paper assessed WNV in an ecological context, disease-modeling efforts would be greatly improved with the inclusion of mosquito infection data. Larson et al. [30] and Diuk-Wasser et al. [31] found that primary WNV vectors tended to be more abundant in rural settings, a finding that could shed light on results found with human incidence in this study. *Culex tarsalis,* the most competent WNV vector in the Great Plains, is primarily located west of the Mississippi River. Although mosquito surveillance is severely lacking in the state, Meece et al. [51] did record *Cx. tarsalis* in the heavily populated southeastern counties, shedding light on possible transmission dynamics from a less common mosquito vector. However, with no mosquito abatement agencies in the state, the only dedicated mosquito control personnel are small, temporary teams hired by city public health for seasonal control. Attempts to gather larval and adult mosquito trap data resulted in only two sources with limited results. 

The positive association of WNV risk with increased air temperature aligns with studies in the Upper Midwestern U.S. and Ontario Province [37,52,53,54]. However, increases in precipitation had mixed effects by species and may be more reliable with the incorporation of timing by temporal lags [54,55,56,57]. The Intergovernmental Panel on Climate Change [58] assessment reports indicate rapid and visible changes to temperature and precipitation globally. Insect vectors, largely mediated by climate, will likely benefit greatly from these predicted scenarios [1]. Several studies have noted warmer winters and fewer days with a hard freeze are strongly associated with increasing WNV cases [59,60,61,62]. Furthermore, these conditions will likely support greater overwintering survival, range expansion, access to novel hosts, earlier seasonal emergences and rapid development rates for WNV mosquito vectors [24,60,63,64,65,66,67]. In addition to these abiotic forces, the continual increases in human population and urbanization will provide insect vectors increased biting opportunities, resulting in higher disease prevalence [68,69,70,71,72]. In this study, both increasing mean monthly temperature and proximity to urban areas were positively associated with disease incidence in nearly every species evaluated.

Disease modeling has limited success predicting disease across large geographic areas. An increasing number of studies are shedding light on the importance of micro and regional habitats in vector-borne disease ecology [11,30,31,73,74]. Due to small-scale regional variations among mosquito and bird species, calculated disease rates are often not generalizable across larger geographic regions. Applying averaged and cumulative monthly environmental predictors may not have captured daily or weekly variability, most notably heavy rains that may wash away mosquito-breeding sites [66], which affect the WNV transmission cycle. As climate regimes continue to shift and the distribution ranges of vectors and disease expand, optimal mosquito-borne disease models will require higher spatiotemporal resolutions of cases and environmental, climatic, and mosquito trap data. 

## 5. Conclusions

This is the first complete assessment of WNV transmission risk factors in Wisconsin, a state with minimal surveillance and research on the mosquito-borne disease.

Increasing temperature and decreasing precipitation, along with urbanization and human population growth, likely favor mosquito biting rates and increase WNV transmission. This study found two key predictors, monthly mean temperature and proximity to urban areas, to be positively associated with increased WNV cases for nearly all species evaluated.

The three most populous counties, Dane, Milwaukee, and Waukesha, contributed the majority of WNV cases for all species. However, controlling for human population and wildlife sampling biases, these counties were found to have relatively low WNV incidence (with the exception of the outbreak years, 2002 and 2012). Overall, the more rural western and northwestern regions of the state were more likely to have increased WNV incidence than any other region for any species.

## Figures and Tables

**Table 1:** Final case status for all specimens tested for WNV in Wisconsin by species type and year. The original case definitions used by the USGS, Wisconsin DHS, Wisconsin DNR, and Wisconsin WVDL are listed in the footnotes.

	Avian					Mammal					Unknown
Year	*Negative*	*Positive*	*Probable*	*Suspect*	*Undetermined*	*Negative*	*Positive*	*Probable*	*Suspect*	*N/A*	*Negative*
2000	3	0	0	0	0	0	0	0	0	0	0
2001	238	2	0	1	129	11	1	0	4	0	0
2002	157	56	0	1	143	583	323	1	23	0	0
2003	2023	190	6	8	28	200	31	0	1	0	0
2004	1706	127	16	4	25	200	33	0	0	0	1
2005	1510	58	0	1	12	156	56	0	2	2	0
2006	3612	156	5	6	5	135	45	5	2	0	0
2007	1436	64	1	0	3	86	29	1	4	0	0
2008	1306	49	2	0	0	53	14	0	0	0	0
2009	619	10	2	6	0	43	2	0	0	0	0
2010	631	11	1	9	6	6	2	0	0	0	0
2011	331	18	0	0	3	14	3	0	0	0	0
2012	1228	41	0	3	11	19	58	1	0	0	0
2013	66	64	1	940	7	20	18	4	0	0	0
2014	56	43	0	403	1	4	6	1	0	0	0
2015	84	56	0	691	4	8	7	2	0	0	0
2016 ^1^	3	0	0	0	0	1	0	0	0	0	0
Unknown	3	3	0	0	0	15	8	3	1	0	0
**Total**	**15012**	**948**	**34**	**2073**	**377**	**1554**	**636**	**18**	**37**	**2**	**1**

USGS: Final confirmed WNV-positive cases were determined based on a diagnostically compatible case that meets the confirmed laboratory diagnostic criterion. Diagnostic description; Clinical signs: lethargy, ataxia, unusual posture, inability to perch or stand, recumbency, and death; Gross necropsy: poor to emaciated body conditions, calvarial or meningeal hemorrhage or congestions, myocardial pallor and splenomegaly; Histopathology: myocardial inflammation, necrosis, and fibrosis. Laboratory criteria: *Confirmed:* Virus isolation in vero cells from feather pulp, kidney/spleen pool, brain or liver. Positive cultures are confirmed as WNV via RT-PCR. *Suspect:* A bird with characteristic gross and histological findings, but negative virus cultures or positive serology. *Present:* Serology positive only in absence of any overt signs of disease. Additional diagnostic comments: positive serology = reactivity ≥1:40. Titer must be 4-fold greater for WNV than SLEV. DHS: All corvid species were submitted to WVDL for PCR analysis (skin tissue specimens). All other avian species (also analyzed by WVDL) were tested for isolation of WNV nucleic acid (positive) and positive immunoglobulin M (IgM) serology (probable). Clinical criteria included death, with/without neurological signs observed (prior to death). Final case definitions for all DHS accessions are confirmed based on the CDC’s arboviral case definition [34]. A positively confirmed human case consisted of clinical criteria (with/without neuroinvasive disease) and isolation of viral nucleic acid from tissue, blood, cerebrospinal fluid (CSF), or other body fluid when testing conducted at the Wisconsin State Laboratory of Hygiene (WSLH). If no isolation of virus is conducted, virus-specific IgM antibodies in CSF or serum are adequate for confirmation. Probable human cases meet clinically compatible criteria (with/without neuroinvasive disease) and with virus-specific IgM antibodies in CSF or serum but with no other testing. DNR: Final case definitions were confirmed based on WNV PCR via one of two reference laboratories (either USGS NWHC or the WVDL). WVDL: All results presented as virus-specific antibody (IgM and/or IgG). Post-mortem equine cases were confirmed WNV positive if brain tissue was PCR positive and histopathology was consistent. Ante-mortem equine cases were confirmed WNV positive if virus-specific IgM antibodies were ELISA positive from serum or CSF. ^1^Data for this study was included through March 2016.

**Table 2:** In total, 1636 submissions tested positive for WNV in Wisconsin from 2001 to 2016, comprising 50 different species (avian: 20 families, 42 species; mammalian: 6 families, 8 species).

Species			Number WNV Positive															
Group	Common Name	Scientific Name	2001	2002	2003	2004	2005	2006	2007	2008	2009	2010	2011	2012	2013	2014	2015	Unk.
Avian	Blackbird, Unidentified	*Icteridae*						1										
	Bluebird, Eastern	*Sialia sialis*			2			1										
	Cardinal, Northern	*Cardinalis cardinalis*		1			2	1									1	
	Chickadee, Black-capped	*Poecile atricapillus*			2													
	Chicken, Greater Prairie	*Tympanuchus cupido*							1		1							
	Cormorant, Double-crested	*Phalacrocorax auritus*			3			1		2		6						
	Corvid, Unidentified	*Corvidae*														2	1	
	Crane, Blue (Stanley or Paradise)	*Anthropoides paradiseus*				1												
	Crane, Hooded	*Grus monacha*				2												
	Crane, Indian Sarus	*Grus antigone antigone*				2												
	Crane, Red-crowned (Japanese)	*Grus japonensis*				2												
	Crane, Sandhill	*Grus canadensis*		3	6												3	
	Crane, Sarus, Unidentified	*Grus antigone*		1														
	Crane, Siberian	*Grus leucogeranus*		1		3												
	Crane, Wattled	*Bugeranus carunculatus*		5		2												
	Crane, White-naped	*Grus vipio*				1												
	Crane, Whooping	*Grus americana*		1		6	6	3	6	1	3	1		4	3	8		
	Crow, American	*Corvus brachyrhynchos*		14	146	109	38	108	43	30	5	3	15	33	53	33	40	
	Dove, Mourning	*Zenaida macroura*		1				1										
	Eagle, Bald	*Haliaeetus leucocephalus*	2	2	10	1		2	3	2				1				1
	Emu	*Dromaius novaehollandiae*		1														
	Finch, Unidentified	*Ardeidae*						1										
	Goshawk, Northern	*Accipiter gentilis*		3	1													
	Grackle, Common	*Quiscalus quiscula*			1													
	Hawk, Cooper’s	*Accipiter cooperii*		1					2						2			
	Hawk, Red-tailed	*Buteo jamaicensis*		2	2	2	1	1									1	
	Hawk, Sharp-shinned	*Accipiter striatus*		1		2												
	Hawk, Unidentified	*Accipitridae*								1								
	Jay, Blue	*Cyanocitta cristata*			18	9	9	28	6	15	2	1	3	3	5		9	
Avian	Loon, Common	*Gavia immer*													1			
	Merlin	*Falco columbarius*		1														
	Owl, Horned, Great	*Bubo virginianus*				1												1
	Pelican, Unidentified	*Pelecanus*																1
	Pelican, White, American	*Pelecanus erythrorhynchos*						8				1						
	Raven, Common	*Corvus corax*						3	1						1		1	
	Robin, American	*Turdus migratorius*			2													
	Sora	*Porzana carolina*					1											
	Sparrow, Unidentified	*Passeridae*						1			1							
	Starling, European	*Sturnus vulgaris*			1													
	Swan, Trumpeter	*Cygnus buccinator*		16					2									
	Swan, Tundra	*Cygnus columbianus*		1														
	Thrush, Unidentified	*Turdidae*							1									
	Turkey, Wild	*Meleagris gallopavo*						1										
	Waxwing, Cedar	*Bombycilla cedrorum*			2													
	Woodpecker, Downy	*Dryobates pubescens*		1														
	Woodpecker, Hairy	*Picoides villosus*			1													
Mammal	Bat, Brown, Big	*Eptesicus fuscus*																
	Bat, Brown, Little	*Myotis lucifugus*																
	Coyote	*Canis latrans*																
	Elk	*Cervus canadensis*			7	1	2							1				
	Horse, Domestic	*Equus ferus caballus*		270	2	19	31	21	16	6	1			1				
	Human	*Homo sapien*		46	18	12	17	23	14	8	1	2	3	57	22	6	9	1
	Squirrel, Gray, Eastern	*Sciurus carolinensis*		6			1									1		
	Unknown	*Unknown*		1														
	Wolf, Gray	*Canis lupus*	1	1	3		5	5										

**Table 3:** Reported Wisconsin human WNV cases by county and year (2002–2015) (incidence per 100,000 people).

	2002	2003	2004	2005	2006	2007	2008	2009	2010	2011	2012	2013	2014	2015	Unknown	Total Cases (n)	Annual Incidence (%) ^b^	Cumulative Incidence (%)
Adams	1															1	0.35	4.88
Ashland													1			1	0.45	6.24
Barron	5										1					6	0.94	13.14
Brown	4	1			1							1 (1)	1	1		10	0.28	3.93
Buffalo				1												1	0.53	7.49
Burnett		1														1	0.47	6.52
Calumet	1															1	0.14	2.02
Chippewa	1	1	1				1									4	0.45	6.34
Clark		1														1	0.21	2.89
Columbia		1														1	0.13	1.77
Dane	1	2	1	3 *	3	3 (1)		1			5	4		1		24	0.34	4.71
Dodge				1		1	2				2					6	0.49	6.79
Douglas		1														1	0.16	2.28
Eau Claire												1 * (1)				2	0.14	1.97
Fond du Lac		1			1											2	0.14	1.96
Grant					2		1				1					4	0.56	7.83
Green	1					1										2	0.39	5.39
Iowa			1		1											2	0.60	8.42
Jefferson	2		1*	2		2			1			1				9	0.76	10.65
Kenosha	2										3	1				6	0.26	3.58
La Crosse					1						3					4	0.24	3.43
Lafayette												2				2	0.85	11.93
Langlade											1*					1	0.36	5.11
Lincoln	1															1	0.25	3.49
Manitowoc	1				1							1				3	0.27	3.72
Marathon				1			1			1				1		4	0.21	2.95
Marinette	1															1	0.17	2.4
Marquette	1															1	0.47	6.59
Milwaukee	9			8 *	7 *	1	1			1	27 ***	3 *	2	3 *		62	0.46	6.49
Oconto										1						1	0.19	2.68
Oneida														1		1	0.2	2.8
Outagamie	1	1	1	1			1 *					1	1 *			7	0.28	3.88
Ozaukee		1														1	0.08	1.15
Polk	1		1								1					3	0.49	6.9
Portage		1	1		1											3	0.3	4.27
Racine	4						1				2			1		8	0.29	4.1
Richland	1															1	0.4	5.64
Rock			1		1	2					2	1				7	0.31	4.35
Rusk	1		1		1											3	1.49	20.84
Shawano					1											1	0.17	2.4
Sheboygan		1														1	0.06	0.87
St. Croix		1				1										2	0.17	2.33
Vernon		1														1	0.24	3.3
Walworth			1		1	2 *						2 (2)		1		9	0.62	8.74
Washington		1							1		1					3	0.16	2.26
Waukesha	5					1					7 *				1	14	0.25	3.55
Winnebago	1	2			1						1		1			6	0.25	3.54
Wood			2													2	0.19	2.7
Unknown	1 *															1	0.09 ^c^	1.28
Total Cases (n)	46	18	12	17	23	14	8	1	2	3	57	18	6	9	1	239	0.3	4.24
Total Deaths (n)	1	0	2	2	1	1	1	0	0	0	5	2	1	1	0	17	0.02	0.3
Annual Incidence	0.8	0.3	0.2	0.3	0.4	0.3	0.1	0	0	0.1	1	0.31	0.1	0.2	0.018 ^a^	0.3	-	-
Cumulative Incidence	0.8	1.2	1.4	1.7	2.1	2.4	2.5	2.5	2.6	2.6	3.6	3.92	4	4.2	4.19	4.24	-	-

^a^ Population based on average from 2002 to 2015 Wisconsin annual values provided by U.S. Census Data; incidence per 100,000 individuals. ^b^ County population values based on most recently provided estimate (year: 2013) by U.S. Census Data. ^c^ Calculation based on average county population (78261) based on 2013 U.S. Census County Data. Values contained within parenthesis indicate probable cases; each asterisk (*) indicates numbers of human deaths. State population values provided from U.S. Census Bureau.

**Table 4:** Spatial relationship for WNV cases in avians, corvids, equines, (controlled by rate) and humans (controlled by incidence) for all years (2001–2016, pooled) and years 2002 and 2012, respectively.

Parameter	Moran’s I	*p*	Spatial Interpretation	Referring Figure
2002 Avians	0.0449	0.486	Random	Figure S1(A1)
* 2002 Corvids	0.159	0.0364	Clustered	Figure S1(B1)
2002 Equines	–0.0212	0.938	Random	Figure S1(C1)
2002 Humans	–0.00128	0.872	Random	Figure S1(D1)
* 2012 Avians	0.201	0.00288	Clustered	Figure S1(B2)
* 2012 Corvids	0.171	0.0264	Clustered	Figure S1(B2)
* 2012 Equines	–0.0463	0.0104	Dispersed	Figure S1(C2)
2012 Humans	0.0914	0.194	Random	Figure S1(D2)
* All Years Avians	0.256	0.00149	Clustered	Figure 3A
* All Years Corvids	0.217	0.00748	Clustered	Figure 3B
* All Years Equines	0.178	0.024	Clustered	Figure 3C
All Years Humans	0.081	0.256	Random	Figure 3D

Spatial autocorrelation indicated by Moran’s I, with resulting significance factor and spatial interpretation (asterisk (*) denotes significant factor for *p* < 0.05).

**Table 5:** Summary of significant parameters (*p* < 0.0001) for statewide WNV modeling, evaluated by linear and logistic regression methods. All factors indicated by an increase (+) or decrease (−) symbol display their respective effect on WNV prevalence.

		*Linear Regression—Statewide*				*Logistic Regression—Statewide*			
Parameters		All Avian Species	Corvids	Equines	Humans	All Avian Species	Corvids	Equines	Humans
Date (mm/yyyy)			+	−					Can Not Assess ^a^
County Population (n)		−	−	+		−	−		
% of County	Agriculture	+	+						
	Forest	+		−					
	Grassland	+	+		+				
	Shrubland	+	+						
	Urban	+	+	−	+	+	+		
	Water	+	+	−					
	Wetland	+	+	+	+				
Average Elevation (ft.)		+	+	−					
Maximum County Temperature (°F)		−	−			+	−		
Total County Accumulation (in.)		+		−	−			−	
Maximum County Daily Accumulation (in.)		−						+	
Mean County Temperature (°F)			+	+		+	+	+	
Average County Daily Accumulation (in.)			−		+	−	−		

^a^ Statewide logistic regression odds ratio based on confirmed cases and non-cases. Binary logistic outcome cannot be tested, as only confirmed human cases were provided for this analysis.

## Supplementary Materials

The following are available online at https://www.mdpi.com/1660-4601/17/5/1767/s1. Table S1: Wisconsin county-specific environmental and land use values, 2001–2016; Table S2: Final WNV case status by species type for each Wisconsin county (cumulative years) (*n* = 72); Figure S1: Hot spot analysis* (A-D1, A-D2) and Empirical Bayesian kriging (A-D3, A-D4) maps displaying the prediction of statewide WNV occurrence for all avian (A), corvid (B), equine (C), and human (D) species for years 2002 (A-D1, A-D3) and 2012 (A-D2, A-D4). * Red areas indicate statistical clustering while blue areas indicated statistical dispersal relationships; Figure S2: Linear regression displaying WNV (human incidence) relationship with increasing human population size by county. The high correlation (r^2^ = 0.724) is indicative of potential selection bias of human subjects.

## Appendix A

**Table A1:** Environmental parameters statewide for years 2001–2016, displaying monthly means for each respective parameter and year combination.

Environmental Parameter	Month	Year															
		2001	2002	2003	2004	2005	2006	2007	2008	2009	2010	2011	2012	2013	2014	2015	2016
Average Accumulation (in.)	*Jan*	0.45	0.48	0.33	0.30	0.39	0.55	0.42	0.36	0.24	0.34	0.30	0.44	0.41	0.27	0.35	0.37
	*Feb*	0.39	0.58	0.37	0.47	0.53	0.42	0.32	0.39	0.47	0.80	0.44	0.54	0.43	0.28	0.28	0.48
	*Mar*	0.55	0.56	0.64	0.70	0.59	0.66	0.71	0.54	0.65	0.74	0.62	0.87	0.54	0.48	0.63	0.81
	*Apr*	0.99	0.92	0.89	0.92	0.94	1.02	0.89	0.93	0.91	1.01	0.89	0.92	0.82	0.89	0.93	0.87
	*May*	1.13	1.01	1.13	1.20	1.02	1.15	1.12	1.03	1.07	1.10	1.04	1.20	1.12	1.08	1.13	1.11
	*Jun*	1.25	1.31	1.19	1.23	1.27	1.22	1.25	1.30	1.19	1.32	1.23	1.24	1.27	1.34	1.23	1.30
	*Jul*	1.27	1.32	1.27	1.26	1.33	1.38	1.28	1.29	1.15	1.41	1.35	1.36	1.24	1.20	1.25	1.34
	*Aug*	1.31	1.27	1.28	1.19	1.24	1.28	1.38	1.19	1.25	1.31	1.23	1.24	1.25	1.30	1.22	1.33
	*Sept*	1.12	1.21	1.13	1.16	1.22	1.07	1.14	1.12	1.12	1.16	1.09	1.10	1.14	1.11	1.21	1.25
	*Oct*	0.89	0.84	0.90	0.95	0.96	0.86	1.03	0.89	0.88	0.96	0.94	0.92	0.91	0.91	0.93	*n*/A
	*Nov*	0.85	0.60	0.69	0.71	0.75	0.69	0.60	0.63	0.73	0.67	0.72	0.67	0.63	0.53	0.82	*n*/A
	*Dec*	0.54	0.49	0.51	0.46	0.41	0.55	0.43	0.42	0.44	0.40	0.52	0.52	0.33	0.47	0.68	*n*/A
Maximum Accumulation (in.)	*Jan*	0.99	0.66	0.70	0.57	0.81	1.02	0.94	0.80	0.51	0.89	0.48	0.70	0.89	0.59	0.59	0.69
	*Feb*	1.01	0.97	0.70	0.81	0.88	0.62	0.90	0.80	0.89	1.42	0.97	1.29	0.93	1.01	0.52	0.84
	*Mar*	0.70	1.05	1.03	1.35	1.19	1.07	1.26	0.83	1.29	1.04	1.39	1.17	0.97	0.99	0.89	1.99
	*Apr*	1.58	1.37	1.67	1.42	1.39	1.35	1.31	1.63	1.57	1.36	1.45	1.56	1.66	1.64	1.44	1.22
	*May*	1.47	1.30	1.69	1.87	1.38	1.62	1.50	1.71	1.42	1.47	1.50	1.73	1.69	1.81	1.90	1.72
	*Jun*	2.20	1.95	1.55	1.79	1.55	1.51	1.75	2.47	1.69	1.84	1.82	1.81	2.04	1.98	1.88	2.16
	*Jul*	1.61	1.91	1.64	1.86	2.10	1.72	2.02	1.99	1.48	2.74	1.76	1.72	1.75	1.61	2.08	1.90
	*Aug*	2.00	1.94	1.57	1.56	1.64	1.95	2.45	1.52	2.09	2.13	1.56	1.49	1.74	1.97	1.81	1.79
	*Sept*	2.02	2.25	1.70	1.82	1.58	1.53	1.55	1.33	1.43	2.08	1.59	1.54	1.53	1.67	1.91	2.29
	*Oct*	1.35	1.73	1.33	1.48	1.99	1.48	1.64	1.43	1.46	1.53	1.49	1.50	1.46	1.90	1.54	*n*/A
	*Nov*	1.26	0.88	1.60	1.02	1.43	1.13	0.78	1.05	0.93	0.95	1.14	1.31	1.26	1.07	1.57	*n*/A
	*Dec*	1.07	0.86	0.99	0.81	0.65	0.98	1.30	0.75	1.22	0.93	0.97	0.88	0.67	0.94	1.91	*n*/A
Total Accumulation (in.)	*Jan*	13.86	14.73	10.23	9.23	12.06	16.98	13.00	11.10	7.53	10.13	9.32	13.74	12.82	8.22	10.72	11.44
	*Feb*	10.86	16.14	10.42	13.67	14.79	11.79	9.00	10.88	13.03	23.88	12.27	15.00	12.10	7.76	7.73	14.28
	*Mar*	17.16	17.21	19.72	21.67	18.17	20.61	22.11	16.87	20.16	23.07	19.08	26.86	16.82	14.90	19.51	25.14
	*Apr*	29.72	27.52	26.65	27.54	28.12	30.48	26.63	28.02	27.22	30.30	26.65	27.66	24.65	27.59	28.03	26.11
	*May*	35.13	31.35	35.11	37.26	31.62	35.68	34.67	31.82	33.04	34.11	32.16	37.08	34.67	33.53	34.90	34.54
	*Jun*	37.42	39.18	35.69	36.94	38.13	36.63	37.47	39.11	35.65	39.47	37.01	37.27	38.07	40.23	36.91	38.92
	*Jul*	39.30	40.98	39.23	39.21	41.10	42.66	39.76	40.05	35.77	43.73	41.80	42.11	38.51	37.09	38.85	41.51
	*Aug*	40.50	39.39	39.58	36.81	38.37	39.77	42.82	36.74	38.74	40.75	38.25	38.49	38.68	40.26	37.76	41.16
	*Sept*	33.84	36.21	33.82	34.74	36.46	32.16	34.07	33.58	33.47	34.82	32.76	33.08	34.23	33.38	36.44	37.56
	*Oct*	27.65	25.96	27.76	29.49	29.65	26.56	31.84	27.55	27.13	29.65	29.16	28.61	28.35	28.10	28.92	*n*/A
	*Nov*	25.55	17.93	20.57	21.24	22.35	20.68	18.12	19.04	21.78	19.99	21.74	20.16	18.93	15.88	24.45	*n*/A
	*Dec*	16.74	15.06	15.67	14.36	12.77	17.14	13.38	13.07	13.78	12.26	16.04	16.11	10.26	14.53	21.01	*n*/A
Mean Temperature (°F)	*Jan*	18.38	22.83	13.27	10.76	13.71	26.38	19.29	14.03	6.21	14.32	11.79	19.97	16.26	5.91	14.72	15.85
	*Feb*	14.69	25.04	13.33	19.84	23.26	17.33	11.03	12.80	19.03	18.55	17.39	24.51	16.04	7.09	7.78	21.29
	*Mar*	26.35	23.86	27.56	31.49	25.91	29.46	32.60	23.85	28.23	34.46	26.27	42.00	22.54	20.67	28.77	34.52
	*Apr*	43.78	39.75	39.49	41.58	44.67	45.37	39.88	40.06	40.37	46.16	38.96	42.11	36.02	37.10	41.51	39.90
	*May*	52.78	47.03	49.94	49.28	48.81	51.97	54.97	48.46	50.48	53.83	50.31	55.49	51.39	51.23	52.51	51.51
	*Jun*	60.28	61.93	57.56	56.99	64.72	60.43	61.92	59.69	59.23	60.78	59.27	62.52	59.70	61.29	59.64	61.37
	*Jul*	65.17	67.21	63.76	61.79	66.36	67.73	64.64	64.09	59.37	66.27	68.57	70.36	63.91	61.44	63.12	66.17
	*Aug*	65.55	62.68	64.70	58.00	63.40	63.05	64.56	62.14	60.73	65.96	63.44	62.84	63.80	62.55	61.42	65.06
	*Sept*	53.48	57.83	55.20	58.22	59.44	53.20	57.37	56.89	57.47	53.04	54.06	53.66	56.17	54.49	60.92	58.48
	*Oct*	43.52	39.63	44.16	45.36	46.78	39.88	49.67	44.07	39.30	46.11	46.20	42.21	44.19	42.26	44.61	*n*/A
	*Nov*	40.59	30.02	30.97	34.87	32.99	34.62	30.88	31.04	37.43	32.76	34.38	32.55	28.93	24.11	36.80	*n*/A
	*Dec*	25.85	23.28	23.76	19.78	17.54	25.72	16.69	12.56	17.84	16.11	24.95	23.75	11.84	23.12	30.01	*n*/A
Maximum Temperature (°F)	*Jan*	37.71	47.94	47.75	37.63	38.75	46.68	44.05	43.21	29.43	37.56	37.89	49.18	45.62	37.59	38.79	39.78
	*Feb*	37.76	47.45	45.81	47.23	48.07	40.23	45.63	36.65	47.01	38.04	49.72	43.55	39.39	41.83	33.4	51.06
	*Mar*	47.55	51.11	62.72	58.04	62.63	56.98	75.06	47.59	62.68	67.36	54.6	78.49	48.81	55.32	63.04	63.02
	*Apr*	76.13	81.61	80.03	75.45	75.37	74.4	79.84	71.96	76.58	78.08	71.97	71.65	72.41	68.88	73.05	73.95
	*May*	81.26	81.05	74.28	75.8	74.42	85.6	84.24	75.81	81.02	88.19	81.44	86.97	83.84	83.56	80.33	79.27
	*Jun*	85.08	85.64	84.03	82.42	88.47	84.83	87.25	82.42	89.65	84.39	90.85	89.88	83.88	84.06	83.64	83.96
	*Jul*	88.08	89.65	84.42	82.09	90.08	91.94	89.97	84.9	81.06	86.47	92.82	96.3	90.95	86.48	86.42	85.52
	*Aug*	91.09	85.24	88.32	78.8	87.75	91.09	87.81	84.34	84.08	87.18	86.91	89.33	90.94	83.51	88.9	83.07
	*Sept*	78.87	85.46	84.32	81.09	85.56	78.1	85.96	85.41	80.33	79.1	85.99	86.73	88.29	80.23	86.25	77.82
	*Oct*	73.73	76.26	75.39	72.21	79.13	75.47	81.91	75.51	62.63	78.8	79.78	72.41	75.62	67.08	76.95	*n*/A
	*Nov*	62.98	57.75	55.83	59.19	59.65	62.6	55.08	70.13	66.11	63.12	58.86	62.55	55.33	54.6	69.39	*n*/A
	*Dec*	58.31	46.4	45.98	46.9	38.73	45.84	38.04	43.29	44.89	43.45	46.35	56.57	41.79	45.07	51.34	*n*/A

**Table A2:** Environmental parameters statewide for years 2001–2016, displaying tests for statistical differences for each respective parameter and year combination.

Source	DF	F	*P*
Average Daily Accumulation for State (in.)			
By: Month/Year			
Model	185	5.58	0.164
Error	2		
Month/Year	185	5.58	0.164
By: Year			
Model	15	0.16	0.999
Error	172		
Year	15	0.30	0.020
Maximum Daily Accumulation for State (in.)			
By: Month/Year			
Model	185	3.28	0.263
Error	2		
Date	185	3.28	0.263
By: Year			
Model	15	0.34	0.991
Error	172		
Year	15	0.34	0.991
Average Total Accumulation for State (in.)			
By: Month/Year			
Model	185	5.92	0.155
Error	2		
Date	185	5.92	0.155
By: Year			
Model	15	0.16	0.999
Error	172		
Year	15	0.16	0.999
Average Temperature for State (°F)			
By: Month/Year			
Model	185	8	0.117
Error	2		
Date	185	8	0.117
By: Year			
Model	15	0.16	0.999
Error	172		
Year	15	0.16	0.999
Maximum Temperature for State (°F)			
By: Month/Year			
Model	185	4.74	0.190
Error	2		
Date	185	4.74	0.190
By: Year			
Model	15	0.15	0.999
Error	172		
Year	15	0.15	0.999

## References

[1] Paz S. (2015). Climate change impacts on West Nile virus transmission in a global context. Philos. Trans. R. Soc. B.

[2] May F.J., Davis C.T., Tesh R.B., Barrett A.D. (2011). Phylogeography of West Nile virus: From the cradle of evolution in Africa to Eurasia, Australia, and the Americas. J. Virol..

[3] Weaver S.C., Reisen W.K. (2010). Present and future arboviral threats. Antivir. Res..

[4] Kramer L.D., Styer L.M., Ebel G.D. (2008). A global perspective on the epidemiology of West Nile virus. Annu. Rev. Entomol..

[5] Petersen L.R., Brault A.C., Nasci R.S. (2013). West Nile virus: Review of the literature. J. Am. Med Assoc..

[6] Centers for Disease Control (2018). National Center for Emerging and Zoonotic Infectious Diseases (NCEZID), Division of Vector-Borne Diseases (DVBD).

[7] American Society for Microbiology (2013). West Nile Virus Frequently Asked Questions.

[8] Hughes T., Irwin P., Hofmeister E., Paskewitz S.M. (2010). Occurrence of avian *Plasmodium* and West Nile virus in *Culex* species in Wisconsin. J. Am. Mosq. Control Assoc..

[9] Abdelrazec A., Lenhart S., Zhu H. (2014). Transmission dynamics of West Nile virus in mosquitoes and corvids and non-corvids. J. Math. Biol..

[10] Guptill S.C., Julian K.G., Campbell G.L., Price S.D., Marfin A.A. (2003). Early-season avian deaths from West Nile virus as warnings of human infection. Emerg. Infect. Dis..

[11] Karki S., Hamer G.L., Anderson T.K., Goldberg T.L., Kitron U.D., Krebs B.L., Walker E.D., Ruiz M.O. (2016). Effect of trapping methods, weather, and landscape on estimates of the *Culex* vector mosquito abundance. Environ. Health Insights.

[12] Tedesco C., Ruiz M.O., Mclafferty S. (2010). Mosquito politics: Local vector control policies and the spread of West Nile Virus in the Chicago region. Health Place.

[13] Sugumaran R., Larson S.R., DeGroote J.P. (2009). Spatio-temporal cluster analysis of county-based human West Nile virus incidence in the continental United States. Int. J. Health Geogr..

[14] Carlton J. (2014). West Nile Virus Hits California—Drought Triggers Skyrocketing Number of Cases—238 in Humans—So Far This Year.

[15] Haines A., Patz J.A. (2004). Health effects of climate change. J. Am. Med. Assoc..

[16] Grossi-Soyster E.N., Cook E.A.J., de Glanville W.A., Thomas L.F., Krystosik A.R., Wamae C.N., Kariuki S., Fevre E.M., LaBeaud A.D. (2017). Serological and spatial analysis of alphavirus and flavivirus prevalence and risk factors in a rural community in western Kenya. PLoS Negl. Trop. Dis..

[17] Anyamba A., Small J.L., Britch S.C., Tucker C.J., Pak E.W., Reynolds C.A., Crutchfield J., Linthicum K.J. (2014). Recent weather extremes and impacts on agricultural production and vector-borne disease outbreak patterns. PLoS ONE.

[18] Shuman E.K. (2010). Global climate change and infectious diseases. Int. J. Occup. Environ. Med..

[19] Ivers L.C., Ryan E.T. (2006). Infectious diseases of severe weather-related and flood-related natural disasters. Curr. Opin. Infect. Dis..

[20] Chevalier V., Tran A., Durand B. (2014). Predictive modeling of West Nile virus transmission risk in the mediterranean basin: How far from landing?. Int. J. Environ. Res. Public Health.

[21] Talbot B., Ardis M., Kulkarni M.A. (2019). Influence of demography, land use, and urban form on West Nile virus risk and human West Nile virus incidence in Ottawa, Canada. Vector-Borne Zoonotic Dis..

[22] Brown H.E., Childs J.E., Diuk-Wasser M.A., Fish D. (2008). Ecological factors associated with West Nile virus transmission, northeastern United States. Emerg. Infect. Dis..

[23] Illinois Department of Public Health (IDPH) (2019). West Nile Virus (WNV) Fact Sheet.

[24] Morin C.W., Comrie A.C. (2013). Regional and seasonal response of a West Nile virus vector to climate change. Proc. Natl. Acad. Sci. USA.

[25] Centers for Disease Control (2017). One Health Basics.

[26] United States Census Bureau (2017). Quick Facts Data Sheet: Wisconsin.

[27] SAS Institute Inc. (2017). Version 9.4..

[28] United States Geological Survey (2017). West Nile Virus Case Definition.

[29] MRCC (2017). Midwestern Regional Climate Center cli-MATE Tool.

[30] Larson S.R., DeGroote J.P., Bartholomay L.C., Sugumaran R. (2010). Ecological niche modeling of potential West Nile virus vector mosquito species in Iowa. J. Insect Sci..

[31] Diuk-Wasser M.A., Brown H.E., Andreadis T.G., Fish D. (2006). Modeling the spatial distribution of mosquito vectors for West Nile virus in Connecticut, USA. J. Vector-Borne Zoonotic Dis..

[32] Dutch S. (2000). Wisconsin Bedrock Elevation Map.

[33] State Cartographer’s Office (2017). University of Wisconsin: Madison, WI, USA. https://www.sco.wisc.edu/2016/09/23/wiscland-2-project-complete-data-now-available/.

[34] Centers for Disease Control (2015). Arboviral Diseases, Neuroinvasive and Non-Neuroinvasive 2015 Case Definition.

[35] Sallam M.F., Fizer C., Pilant A.N., Whung P.-Y. (2017). Systematic review: Land cover, meteorological, and socioeconomic determinants of *Aedes* mosquito habitat for risk mapping. Int. J. Environ. Res. Public Health.

[36] Akaike H. (1974). A new look at the statistical model identification. IEEE Trans. Autom. Control.

[37] Environmental Systems Research Institute (ESRI) (2017). ArcGIS Desktop: Release 10.5.1.

[38] Giordano B.V., Kaur S., Hunter F.F. (2017). West Nile virus in Ontario, Canada: A twelve-year analysis of human case prevalence, mosquito surveillance, and climate data. PLoS ONE.

[39] Boehringer-Ingelheim Vetera®. https://www.boehringer-ingelheim.com/animal-health/companion-animals-products/vetera?.

[40] DVM360 (2004). Merial Unveils Equine WNV Vaccine.

[41] Gates M.C., Boston R.C. (2009). Irrigation linked to a greater incidence of human and veterinary West Nile virus cases in the United States from 2004 to 2006. Prev. Vet. Med..

[42] DeGroote J.P., Sugumaran R., Brend S.M., Tucker B.J., Bartholomay L.C. (2008). Landscape, demographic, entomological, and climatic associations with human disease incidence of West Nile virus in the state of Iowa, USA. Int. J. Health Geogr..

[43] Wimberly M.C., Hildreth M.B., Boyte S.P., Lindquist E., Kightlinger L. (2008). Ecological niche of the 2003 West Nile virus epidemic in the northern Great Plains of the United States. PLoS ONE.

[44] Harrigan R.J., Thomassen H.A., Buermann W., Smith T.B. (2014). A continental risk assessment of West Nile virus under climate change. Glob. Chang. Biol..

[45] LaDeau S.L., Kilpatrick A.M., Marra P.P. (2007). West Nile virus emergence and large-scale declines of North American bird populations. Nature.

[46] VanDalen K.K., Hall J.S., Clark L., McLean R.G., Smeraski C. (2013). West Nile virus infection in American robin: New insights on dose response. PLoS ONE.

[47] Hamer G.L., Chaves L.F., Anderson T.K., Kitron U.D., Brawn J.D., Ruiz M.O., Loss S.R., Walker E.D., Goldberg T.L. (2011). Fine-scale variation in vector host use and force of infection drive localized patterns of West Nile virus transmission. PLoS ONE.

[48] Kilpatrick M.A. (2011). Globalization, land use, and the invasion of West Nile virus. Science.

[49] Hamer G.L., Kitron U.D., Goldberg T.L., Brawn J.D., Loss S.R., Ruiz M.O., Hayes D.B., Walker E.D. (2009). Host selection by *Culex pipiens* mosquitoes and West Nile virus amplification. Am. J. Trop. Med. Hyg..

[50] Janousek W.M., Marra P.P., Kilpatrick A.M. (2014). Avian roosting behavior influences vector-host interactions for West Nile virus hosts. Parasites Vectors.

[51] Meece J.K., Henkel J.S., Glaser L., Reed K.D. (2003). Mosquito surveillance for West Nile virus in Southeastern Wisconsin—2002. Clin. Med. Res..

[52] Karki S., Brown W.M., Uelmen J.A., Ruiz M.O., Smith R.L. (2019). The Drives of West Nile Virus Human Illness: Fine Scale Dynamic Effects of Weather, Mosquito Infection, Social, and Biological Conditions.

[53] Hahn M.B., Monaghan A.J., Hayden M.H., Eisen R.J., Delorey M.J., Lindsey N.P., Nasci R.S., Fischer M. (2015). Meteorological conditions associated with increased incidence of West Nile virus disease in the United States, 2004–2012. Am. J. Trop. Med. Hyg..

[54] Ruiz M.O., Chaves L.F., Hamer G.L., Sun T., Brown W.M., Walker E.D., Haramis L., Goldberg T.L., Kitron U.D. (2010). Local impact of temperature and precipitation on West Nile virus infection in *Culex* species mosquitoes in northeast Illinois, USA. Parasites Vectors.

[55] Karki S., Westcott N.E., Muturi E.J., Brown W.M., Ruiz M.O. (2018). Assessing human risk of illness with West Nile virus mosquito surveillance data to improve public health preparedness. Zoonoses Public Health.

[56] Davis J.K., Vincent G., Hildreth M.B., Kightlinger L., Carlson C., Wimberly M.C. (2017). Integrating environmental monitoring and mosquito surveillance to predict vector-borne disease: Prospective forecasts of a West Nile virus outbreak. PLoS Curr. Outbreaks.

[57] Wimberly M.C., Giacomo P., Kightlinger L., Hildreth M.B. (2013). Spatio-temporal epidemiology of human West Nile virus disease in South Dakota. Int. J. Environ. Res. Public Health.

[58] Intergovernmental Panel on Climate Change (2007). IPCC Fourth Assessment Report: Climate Change.

[59] Chung W.M., Buseman C.M., Joyner S.N., Hughes S.M., Fomby T.B., Luby J.P., Haley R.W. (2013). The 2012 West Nile encephalitis epidemic in Dallas, Texas. J. Am. Med. Assoc..

[60] Paz S., Semenza J.C. (2013). Environmental drivers of West Nile fever epidemiology in Europe and Western Asia: A review. Int. J. Environ. Res. Public Health.

[61] Reisen W.K., Thiemann T., Barker C.M., Lu H., Carroll B., Fang Y., Lothrop H.D. (2010). Effects of warm winter temperature on the abundance and gonotrophic activity of *Culex* (Diptera: Culicidae) in California. J. Med. Entomol..

[62] Epstein P.R. (2001). Climate change and emerging infectious diseases. Microbes Infect..

[63] Uelmen J.A., Lindroth R.L., Tobin P.C., Reich P.B., Schwartzberg E.G., Raffa K.F. (2016). Effects of winter temperatures, spring degree-day accumulation, and insect population source on phenological synchrony between forest tent caterpillar and host trees. For. Ecol. Manag..

[64] Patz J.A., Frumkin H., Holloway T., Vimont D.J., Haines A. (2014). Climate change: Challenges and opportunities for global health. J. Am. Med. Assoc..

[65] Fischer D., Thomas S.M., Suk J.E., Sudre B., Hess A., Tjaden N.B., Beierkuhnlein C., Semenza J.C. (2013). Climate change effects on Chikungunya transmission in Europe: Geospatial analysis of vector’s climatic suitability and virus’ temperature requirements. Int. J. Health Geogr..

[66] Chuang T.-W., Wimberly M.C. (2012). Remote sensing of climatic anomalies and West Nile virus incidence in the Northern Great Plains of the United States. PLoS ONE.

[67] Lafferty K.D. (2009). The ecology of climate change and infectious diseases. Ecology.

[68] Kilpatrick M.A., Randolph S.E. (2012). Drivers, dynamics, and control of emerging vector-borne zoonotic diseases. Lancet.

[69] Deichmester J.M., Telang A. (2011). Abundance of West Nile virus mosquito vectors in relation to climate and landscape variables. J. Vector Ecol..

[70] Tabachnick W.J. (2009). Challenges in predicting climate and environmental effects on vector-borne disease episystems in a changing world. J. Exp. Biol..

[71] Sutherst R.W. (2004). Global change and human vulnerability to vector-borne diseases. Clin. Microbiol. Rev..

[72] Knudsen A.B., Slooff R. (1992). Vector-borne disease problems in rapid urbanization: New approaches to vector control. Bull. World Health Organ..

[73] Myer M.H., Campbell S.R., Johnston J.M. (2017). Spatiotemporal modeling of ecological and sociological predictors of West Nile virus in Suffolk County, NY, mosquitoes. Ecosphere.

[74] Wimberly M.C., Lamsal A., Giacomo P., Chuang T.-W. (2014). Regional variations of climatic influences on West Nile virus outbreaks in the United States. Am. J. Trop. Med. Hyg..

