# Supplementary material for: A 15 Year Evaluation of West Nile Virus in Wisconsin: Effects on Wildlife and Human Health
Source: Int J Environ Res Public Health. 2020 Mar 9;17(5):1767. doi: 10.3390/ijerph17051767 (PMC7084944; doi:10.3390/ijerph17051767)
Supplement: Supplementary file 1 [file ijerph-17-01767-s001.pdf]

Supplemental Materials

Supplemental tables

Table S1. Wisconsin county-specific environmental and land use values, 2001-2016.

County	Dominant Land Use	Average Elevation (ft.)	County Population (n)	Human Density (/mi.^{2})	Water (%)	Forest (%)	Urban (%)	Grassland (%)	Agriculture (%)	Shrubland (%)	Wetland (%)
Adams	Agriculture	826.15	20875	30.32	0.06	0.00	0.00	0.00	1.00	0.00	0.00
Ashland	Forest	1116.79	16157	7.05	0.54	0.57	0.05	0.00	0.00	0.00	0.28
Barron	Forest	1006.52	45870	51.55	0.03	0.12	0.02	0.00	0.76	0.00	0.18
Bayfield	Forest	933.50	15014	7.35	0.28	0.82	0.01	0.03	0.00	0.00	0.18
Brown	Agriculture	658.33	248007	402.80	0.14	0.00	0.21	0.00	0.72	0.00	0.08
Buffalo	Shrubland	800.61	13587	19.15	0.05	0.00	0.00	0.00	0.36	0.64	0.00
Burnett	Wetland	792.48	15457	17.56	0.07	0.42	0.00	0.00	0.16	0.00	0.43
Calumet	Agriculture	772.59	48971	123.33	0.20	0.00	0.00	0.00	0.85	0.00	0.03
Chippewa	Agriculture	921.46	62415	59.94	0.03	0.01	0.04	0.00	0.72	0.00	0.30
Clark	Agriculture	1042.39	34931	28.93	0.01	0.00	0.00	0.00	0.99	0.00	0.00
Columbia	Agriculture	786.15	56833	71.40	0.04	0.01	0.00	0.00	0.84	0.00	0.13
Crawford	Shrubland	845.66	16644	27.78	0.05	0.00	0.02	0.06	0.00	0.91	0.00
Dane	Agriculture	842.84	487948	394.07	0.03	0.00	0.10	0.10	0.76	0.07	0.00
Dodge	Agriculture	816.11	88558	97.57	0.03	0.00	0.02	0.00	0.93	0.00	0.07
Door	Agriculture	664.31	27785	11.72	0.80	0.00	0.04	0.00	0.67	0.00	0.20
Douglas	Wetland	908.32	44159	29.84	0.12	0.22	0.02	0.06	0.00	0.00	0.66
Dunn	Agriculture	796.87	44352	51.69	0.02	0.18	0.02	0.00	0.77	0.00	0.00
Eau Claire	Agriculture	917.20	98736	153.02	0.01	0.00	0.09	0.00	0.91	0.00	0.00
Florence	Forest	1288.29	4423	8.89	0.02	0.84	0.00	0.00	0.00	0.16	0.01
Fond du Lac	Agriculture	874.20	101633	132.71	0.06	0.00	0.06	0.00	0.94	0.00	0.01
Forest	Forest	1439.04	9304	8.89	0.03	0.58	0.00	0.00	0.00	0.00	0.42
Grant	Shrubland	855.61	51208	43.27	0.03	0.00	0.00	0.16	0.33	0.00	0.00
Green	Agriculture	804.79	36670	62.91	0.00	0.00	0.00	0.31	0.63	0.00	0.00
Green Lake	Agriculture	681.79	19051	50.09	0.08	0.00	0.00	0.00	0.73	0.00	0.38
Iowa	Grassland	925.35	23687	30.85	0.01	0.00	0.00	0.47	0.17	0.38	0.00
Iron	Wetland	1370.83	5916	6.44	0.18	0.41	0.00	0.00	0.00	0.00	0.61
Jackson	Agriculture	918.57	20449	20.44	0.01	0.00	0.00	0.00	1.00	0.00	0.00
Jefferson	Agriculture	692.95	83443	143.14	0.05	0.02	0.05	0.00	1.00	0.00	0.01
Juneau	Agriculture	873.49	26664	33.16	0.05	0.00	0.00	0.00	0.85	0.19	0.00
Kenosha	Agriculture	573.27	166426	220.72	0.64	0.02	0.09	0.00	0.91	0.00	0.00
Kenosha	Agriculture	573.27	166426	220.72	0.64	0.02	0.09	0.00	0.91	0.00	0.00
Kewaunee	Agriculture	663.92	20574	18.97	0.68	0.00	0.00	0.00	1.00	0.00	0.00
La Crosse	Agriculture	831.40	114638	238.87	0.06	0.00	0.11	0.00	0.87	0.04	0.00
Lafayette	Agriculture	839.32	16836	26.53	0.00	0.00	0.00	0.33	0.69	0.00	0.00
Langlade	Forest	1346.91	19977	22.50	0.02	0.61	0.02	0.00	0.04	0.00	0.35
Lincoln	Forest	1278.55	28743	31.69	0.03	0.52	0.02	0.00	0.24	0.00	0.28
Manitowoc	Agriculture	693.13	81442	54.51	0.61	0.00	0.04	0.00	0.91	0.00	0.06
Marathon	Agriculture	1176.44	134063	85.05	0.02	0.02	0.08	0.00	0.81	0.00	0.10
Marinette	Forest	762.64	41749	26.93	0.10	0.44	0.01	0.00	0.15	0.00	0.41

County	Dominant Land Use	Average Elevation (ft.)	County Population (n)	Human Density (/mi.2)	Water (%)	Forest (%)	Urban (%)	Grassland (%)	Agriculture (%)	Shrubland (%)	Wetland (%)
Marquette	Agriculture	629.57	15404	33.18	0.02	0.00	0.00	0.00	0.89	0.00	0.17
Menominee	Forest	903.62	4232	11.60	0.02	1.06	0.00	0.00	0.00	0.00	0.00
Milwaukee	Urban	666.58	947735	796.72	0.80	0.00	0.93	0.00	0.13	0.00	0.00
Monroe	Agriculture	993.01	44673	49.19	0.01	0.00	0.00	0.01	0.77	0.23	0.00
Oconto	Agriculture	687.03	37660	32.78	0.13	0.40	0.00	0.00	0.40	0.00	0.23
Oneida	Wetland	1348.40	35998	29.12	0.10	0.44	0.00	0.00	0.00	0.00	0.68
Outagamie	Agriculture	661.08	176695	274.12	0.01	0.00	0.01	0.00	0.75	0.00	0.18
Ozaukee	Agriculture	662.65	86395	77.40	0.79	0.00	0.22	0.00	0.78	0.00	0.00
Pepin	Agriculture	756.76	7469	30.03	0.07	0.00	0.00	0.00	1.10	0.01	0.00
Pierce	Agriculture	911.11	41019	69.30	0.03	0.00	0.03	0.00	1.04	0.00	0.00
Polk	Agriculture	928.47	44205	46.22	0.04	0.26	0.00	0.00	0.61	0.00	0.15
Portage	Agriculture	933.98	70019	85.11	0.03	0.00	0.02	0.06	0.90	0.00	0.04
Price	Wetland	1281.57	14159	11.08	0.02	0.26	0.00	0.00	0.00	0.00	0.74
Racine	Agriculture	619.34	195408	246.69	0.58	0.09	0.07	0.00	0.83	0.00	0.00
Richland	Shrubland	940.51	18021	30.58	0.01	0.00	0.00	0.17	0.01	0.83	0.00
Rock	Agriculture	710.40	160331	220.81	0.01	0.00	0.05	0.00	0.91	0.00	0.00
Rusk	Wetland	1040.15	14755	15.85	0.02	0.18	0.00	0.00	0.26	0.00	0.54
Sauk	Agriculture	850.08	61976	73.03	0.02	0.10	0.02	0.00	0.44	0.44	0.00
Sawyer	Wetland	1159.70	16557	12.26	0.07	0.39	0.00	0.00	0.03	0.00	0.68
Shawano	Wetland	837.62	41949	46.12	0.02	0.09	0.02	0.00	0.59	0.00	0.27
Sheboygan	Agriculture	696.25	115507	90.86	0.60	0.00	0.13	0.00	0.82	0.00	0.05
St. Croix	Agriculture	967.08	84345	114.65	0.02	0.00	0.03	0.00	0.90	0.00	0.00
Taylor	Forest	1222.31	20689	21.02	0.01	0.38	0.01	0.00	0.32	0.00	0.32
Trempealeau	Agriculture	849.91	28816	38.84	0.01	0.00	0.00	0.00	1.00	0.04	0.00
Vernon	Shrubland	1055.59	29773	36.47	0.03	0.00	0.00	0.15	0.10	0.79	0.00
Vilas	Forest	1422.47	21430	21.05	0.16	0.76	0.00	0.00	0.00	0.00	0.42
Walworth	Agriculture	693.81	102228	177.31	0.04	0.17	0.00	0.00	0.87	0.00	0.00
Washburn	Wetland	965.87	15911	18.65	0.07	0.32	0.00	0.00	0.10	0.00	0.67
Washington	Agriculture	834.66	131887	302.68	0.01	0.00	0.21	0.00	0.83	0.00	0.00
Waukesha	Agriculture	719.17	389891	671.54	0.05	0.13	0.41	0.00	0.51	0.00	0.00
Waupaca	Agriculture	749.45	52410	68.49	0.02	0.00	0.00	0.00	0.77	0.00	0.25
Waushara	Agriculture	798.11	24496	38.43	0.02	0.00	0.00	0.00	0.91	0.00	0.09
Winnebago	Agriculture	678.93	166994	288.63	0.25	0.00	0.32	0.00	0.46	0.00	0.14
Wood	Agriculture	955.67	74749	92.35	0.02	0.00	0.06	0.00	0.96	0.00	0.03
**Grand Total (Average)**	**Agriculture**	**891.00**	**80184**	**99.24**	**0.14**	**0.15**	**0.05**	**0.03**	**0.57**	**0.06**	**0.15**

Table S2: Final WNV case status by species type for each Wisconsin county (cumulative years) (n = 72).

County	Negative				Positive			Probable			Suspect			Undetermined			
	Avian	*Mammal*	*Unknown*	*Total*	*Avian*	*Mammal*	*Total*	*Avian*	*Mammal*	*Total*	*Avian*	*Mammal*	*Total*	*Avian*	*Mammal*	*Total*	*Grand Total*
Adams	93	4		97	18	2	20				20		20	2		2	139
Ashland	57	59		116	2	12	14				4		4				134
Barron	143	4		147	14	11	25				23		23	1		1	196
Bayfield	96	38		134	11	1	12		1	1	15		15				162
Brown	493	6		499	24	10	34		1	1	47		47	3		3	584
Buffalo	38			38	4	1	5										43
Burnett	83	10		93	16	1	17		3	3	3		3				116
Calumet	2	1		3	2	1	3				7		7				13
Chippewa	122	4		126	22	9	31				34		34				191
Clark	53	12		65	10	6	16				10		10	1		1	92
Columbia	242	9		251	12	1	13				11	1	12	2		2	278
Crawford	18	3		21	3	2	5				3	1	4				30
Dane	2769	30		2799	47	28	75		1	1	305		305	7		7	3187
Dodge	239	3		242	13	9	22				33		33	4		4	301
Door	159	3		162	19		19				8		8	5		5	194
Douglas	99	14		113	8	5	13		2	2	8		8				136
Dunn	76	1		77	19	2	21				13		13				111
Eau Claire	326	7		333	32	1	33		2	2	50	1	51				419
Florence	8			8							1		1				9
Fond du Lac	187	7	1	195	16	2	18				28		28				241
Forest	31	4		35	7		7				7		7				49
Grant	81	8		89	7	8	15				3		3				107
Green	61	9		70	12	6	18				15		15				103
Green Lake	30	2		32	5	1	6				4		4				42
Iowa	63	7		70	5	2	7				8		8	1		1	86
Iron	27	5		32	7		7				2		2				41
Jackson	24	10		34	8	3	11				3	3	6				51
Jefferson	141	9		150	10	12	22				62		62				234
Juneau	157	5		162	34	2	36	1		1	16	3	19	6		6	224
Kenosha	188	7		195	18	6	24				30		30				249
Kewaunee	42	2		44	1		1				4		4				49
La Crosse	183	5		188	21	5	26				15		15	12		12	241
Lafayette	17	1		18		2	2				5		5				25
Langlade	41	2		43	7	1	8				16		16	4		4	71
Lincoln	103	9		112	8	1	9				15		15				136
Manitowoc	96	7		103	5	4	9				11		11				123

County	Negative				Positive			Probable			Suspect			Undetermined			
	Avian	*Mammal*	*Unknown*	*Total*	*Avian*	*Mammal*	*Total*	*Avian*	*Mammal*	*Total*	*Avian*	*Mammal*	*Total*	*Avian*	*Mammal*	*Total*	*Grand Total*
Marathon	263	10		273	17	8	25				71		71				369
Marinette	96	6		102	12	1	13	1		1	14		14	1		1	131
Marquette	58	2		60	13	2	15				7		7				82
Menominee	13			13	1		1				1		1				15
Milwaukee	2018	3		2021	32	68	100				186		186	1		1	2308
Monroe	69	6		75	11	2	13				7		7				95
Multiple Counties		13		13													13
Oneida	258	2		260	14	1	15		1	1	42		42				318
Outagamie	678	7		685	30	7	37				68		68				790
Ozaukee	170	4		174	21	1	22				27		27				223
Pepin	13	3		16	6	1	7										23
Pierce	62	3		65	9		9				9		9				83
Polk	73			73	7	3	10				5	1	6				89
Portage	222	7		229	25	3	28				65		65				322
Price	60	5		65	8		8				15		15				88
Racine	374	7		381	14	12	26				58		58				465
Richland	29	3		32	2	1	3										35
Rock	399	10		409	19	11	30	1		1	51		51				491
Rusk	20	4		24	4	3	7				10		10	1		1	42
Sauk	321	8		329	30		30	16		16	22		22	314[a]		314	711
Sawyer	82	19		101	8	1	9				16		16				126
Shawano	86	2		88	8	3	11				8		8				107
Sheboygan	203	3		206	19	1	20				19		19				245
St. Croix	151	6		157	13	4	17				12		12				186
Taylor	31	7		38	3	1	4				3		3				45
Trempealeau	32	3		35	5	2	7				3		3	11		11	56
Vernon	17	10		27	4	1	5				1		1				33
Vilas	152	2		154	13		13				38		38				205
Walworth	173	8		181	16	9	25		3	3	21	1	22				231
Washburn	72	3		75	8	1	9				8		8				92
Washington	311	7		318	17	3	20				42		42				380
Waukesha	970	10		980	32	17	49	10	1	11	195		195				1235
Waupaca	109	11		120	10		10				27		27				157
Waushara	52	2		54	6	2	8				8		8				70
Winnebago	477	7		484	29	6	35		1	1	76		76	1		1	597
Wood	239	59		298	22	3	25				46	1	47				370
Unknown	22	981		1003	6	300	306	5	2	7	41	25	66		2	2	1384
**Grand Total**	**15012**	**1554**	**1**	**16567**	**948**	**636**	**1584**	**34**	**18**	**52**	**2073**	**37**	**2110**	**377**	**2**	**377**	**20692**

Supplemental Figure Legends

Figure S1: Hot spot analysis* (A-D1, A-D2) and Empirical Bayesian kriging (A-D3, A-D4) maps displaying the prediction of statewide WNV occurrence for all avian (A), corvid (B), equine (C), and human (D) species for years 2002 (A-D1, A-D3) and 2012 (A-D2, A-D4). *Red areas indicate statistical clustering while blue areas indicated statistical dispersal relationships.

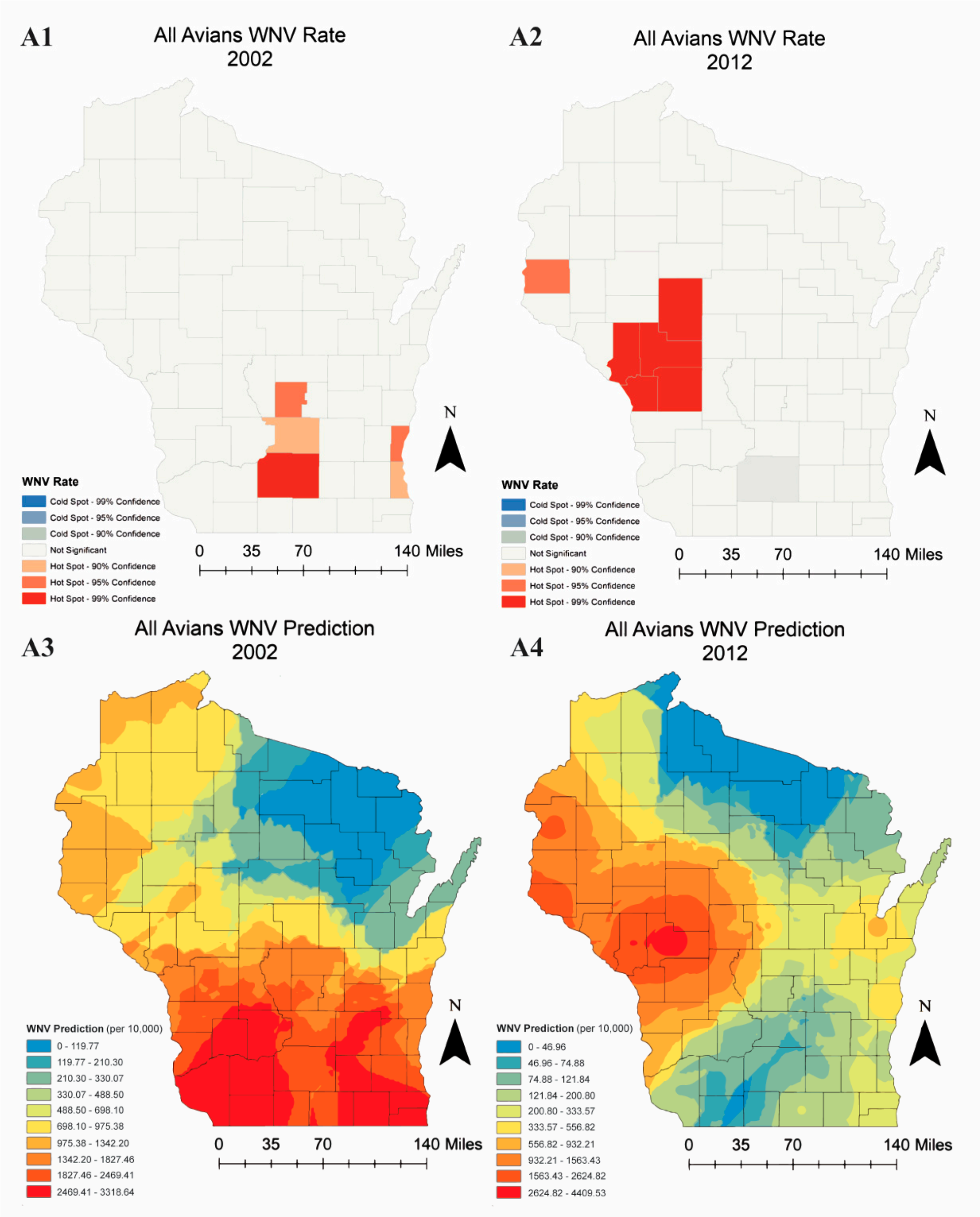
A1
All Avians WNV Rate
2002
WNV Rate
Cold Spot - 99% Confidence
Cold Spot - 95% Confidence
Cold Spot - 90% Confidence
Not Significant
Hot Spot - 90% Confidence
Hot Spot - 95% Confidence
Hot Spot - 99% Confidence
N
0 35 70 140 Miles
A2
All Avians WNV Rate
2012
WNV Rate
Cold Spot - 99% Confidence
Cold Spot - 95% Confidence
Cold Spot - 90% Confidence
Not Significant
Hot Spot - 90% Confidence
Hot Spot - 95% Confidence
Hot Spot - 99% Confidence
N
0 35 70 140 Miles
A3
All Avians WNV Prediction
2002
WNV Prediction (per 10,000)
0 - 119.77
119.77 - 210.30
210.30 - 330.07
330.07 - 488.50
488.50 - 698.10
698.10 - 975.38
975.38 - 1342.20
1342.20 - 1827.46
1827.46 - 2469.41
2469.41 - 3318.64
N
0 35 70 140 Miles
A4
All Avians WNV Prediction
2012
WNV Prediction (per 10,000)
0 - 46.96
46.96 - 74.88
74.88 - 121.84
121.84 - 200.80
200.80 - 333.57
333.57 - 556.82
556.82 - 932.21
932.21 - 1563.43
1563.43 - 2624.82
2624.82 - 4409.53
N
0 35 70 140 Miles

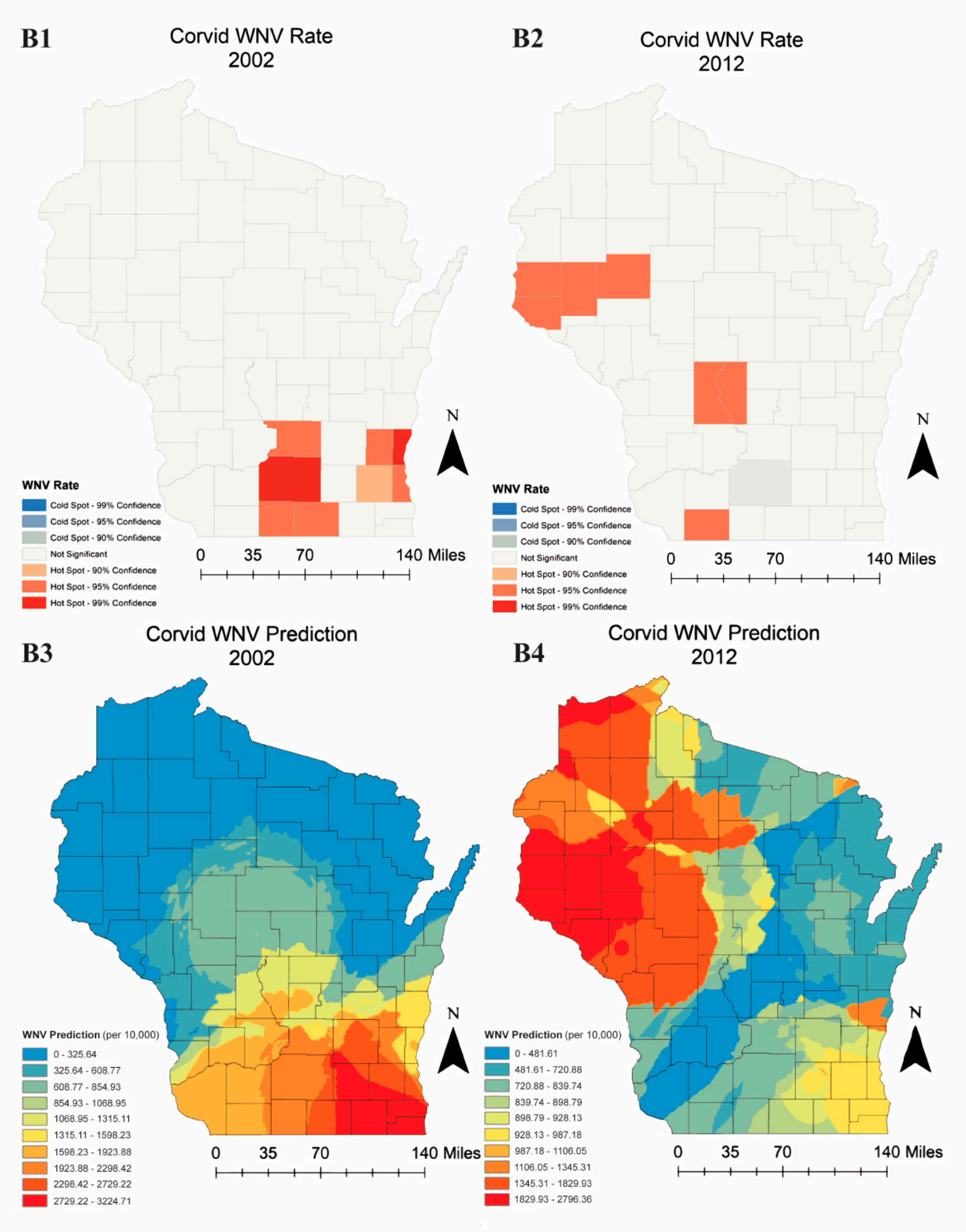
B1
Corvid WNV Rate
2002
WNV Rate
Cold Spot - 99% Confidence
Cold Spot - 95% Confidence
Cold Spot - 90% Confidence
Not Significant
Hot Spot - 90% Confidence
Hot Spot - 95% Confidence
Hot Spot - 99% Confidence
0 35 70 140 Miles
N
B2
Corvid WNV Rate
2012
WNV Rate
Cold Spot - 99% Confidence
Cold Spot - 95% Confidence
Cold Spot - 90% Confidence
Not Significant
Hot Spot - 90% Confidence
Hot Spot - 95% Confidence
Hot Spot - 99% Confidence
0 35 70 140 Miles
N
B3
Corvid WNV Prediction
2002
WNV Prediction (per 10,000)
0 - 325.64
325.64 - 608.77
608.77 - 854.93
854.93 - 1068.95
1068.95 - 1315.11
1315.11 - 1598.23
1598.23 - 1923.88
1923.88 - 2298.42
2298.42 - 2729.22
2729.22 - 3224.71
0 35 70 140 Miles
N
B4
Corvid WNV Prediction
2012
WNV Prediction (per 10,000)
0 - 481.61
481.61 - 720.88
720.88 - 839.74
839.74 - 898.79
898.79 - 928.13
928.13 - 987.18
987.18 - 1106.05
1106.05 - 1345.31
1345.31 - 1829.93
1829.93 - 2796.36
0 35 70 140 Miles
N

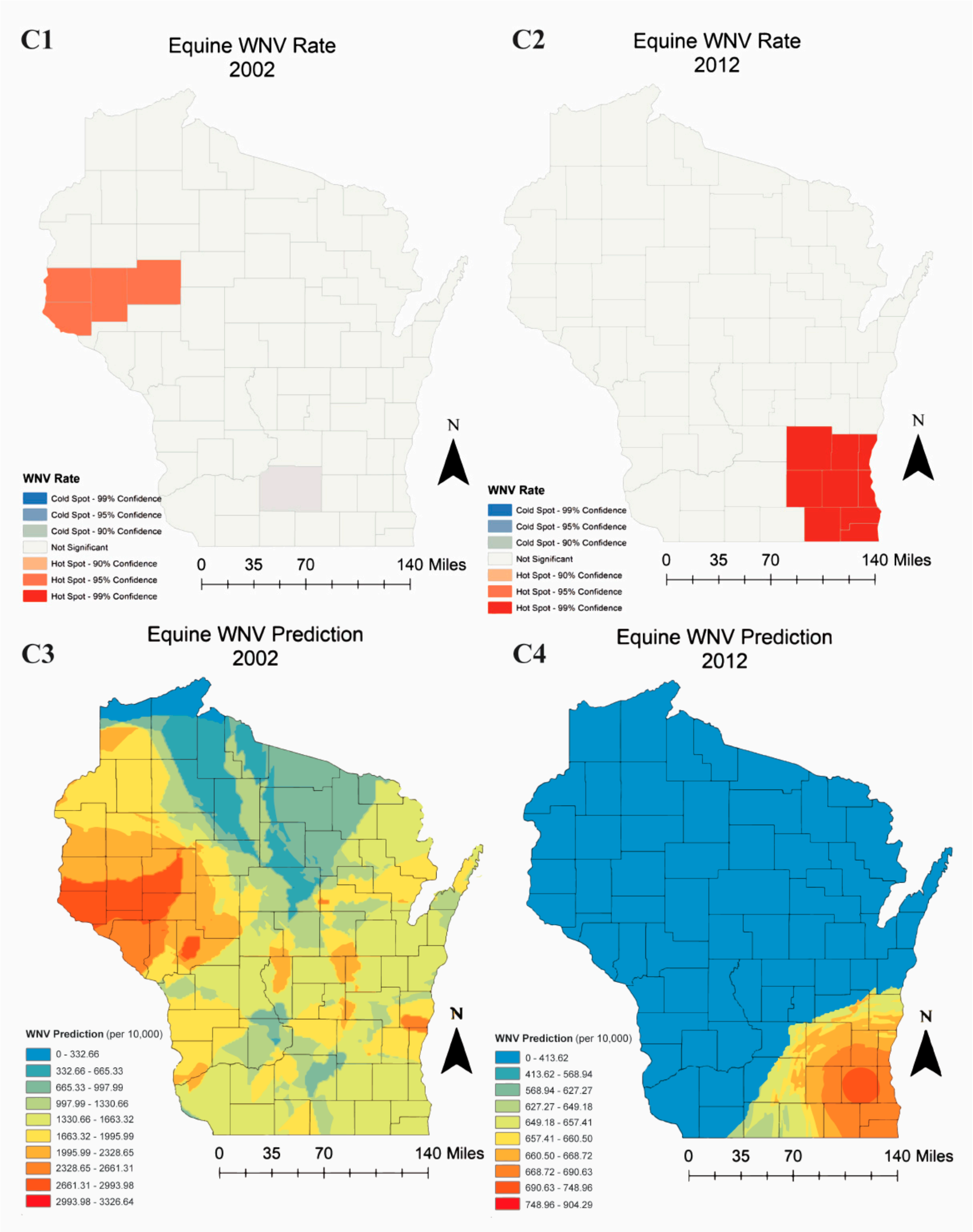
C1
Equine WNV Rate
2002
WNV Rate
Cold Spot - 99% Confidence
Cold Spot - 95% Confidence
Cold Spot - 90% Confidence
Not Significant
Hot Spot - 90% Confidence
Hot Spot - 95% Confidence
Hot Spot - 99% Confidence
0 35 70 140 Miles
N
C2
Equine WNV Rate
2012
WNV Rate
Cold Spot - 99% Confidence
Cold Spot - 95% Confidence
Cold Spot - 90% Confidence
Not Significant
Hot Spot - 90% Confidence
Hot Spot - 95% Confidence
Hot Spot - 99% Confidence
0 35 70 140 Miles
N
C3
Equine WNV Prediction
2002
WNV Prediction (per 10,000)
0 - 332.66
332.66 - 665.33
665.33 - 997.99
997.99 - 1330.66
1330.66 - 1663.32
1663.32 - 1995.99
1995.99 - 2328.65
2328.65 - 2661.31
2661.31 - 2993.98
2993.98 - 3326.64
0 35 70 140 Miles
N
C4
Equine WNV Prediction
2012
WNV Prediction (per 10,000)
0 - 413.62
413.62 - 568.94
568.94 - 627.27
627.27 - 649.18
649.18 - 657.41
657.41 - 660.50
660.50 - 668.72
668.72 - 690.63
690.63 - 748.96
748.96 - 904.29
0 35 70 140 Miles
N

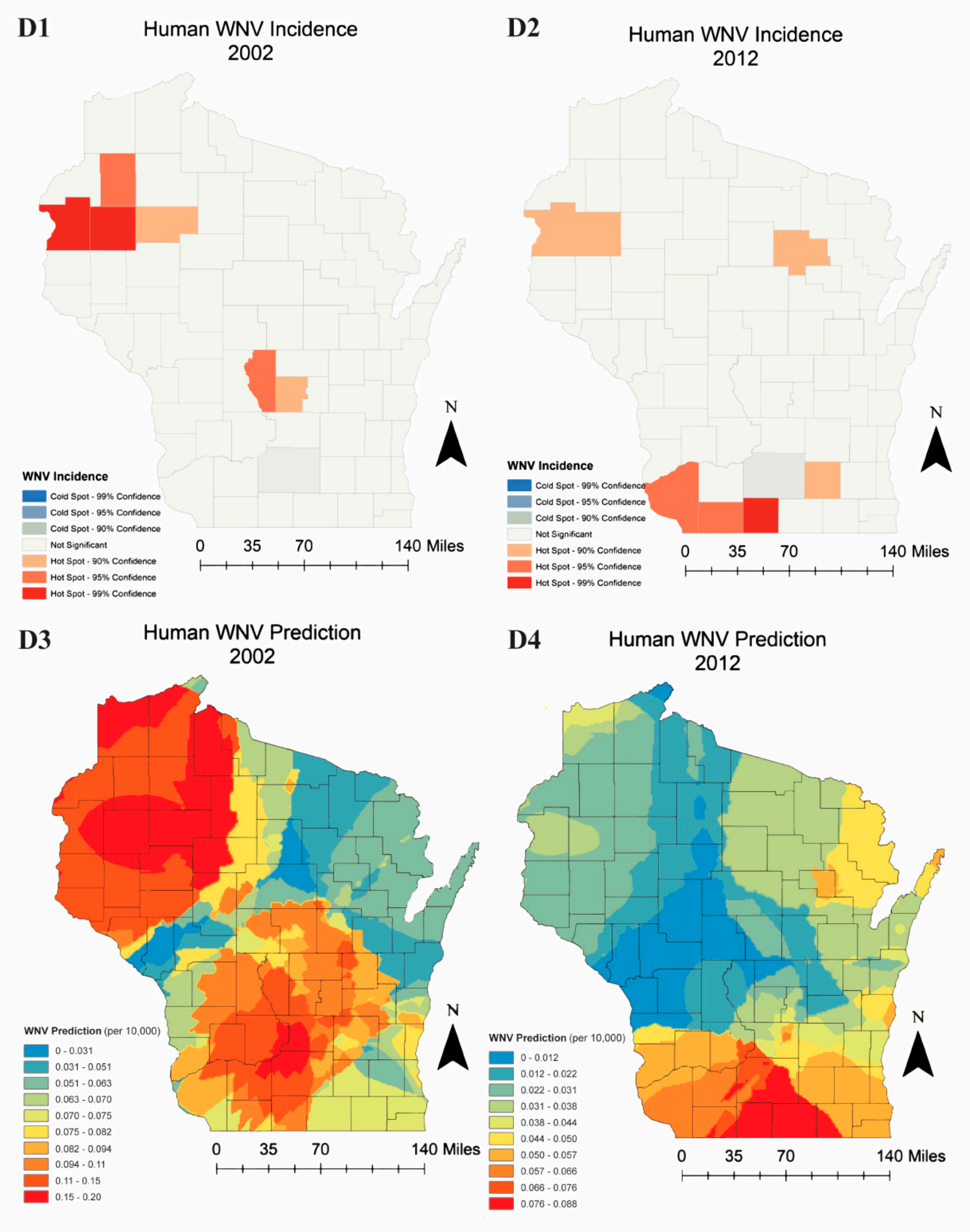

Figure S2. Linear regression displaying WNV (human incidence) relationship with increasing human population size by county. The high correlation ($r^2 = 0.724$) indicative of potential selection bias of human subjects.

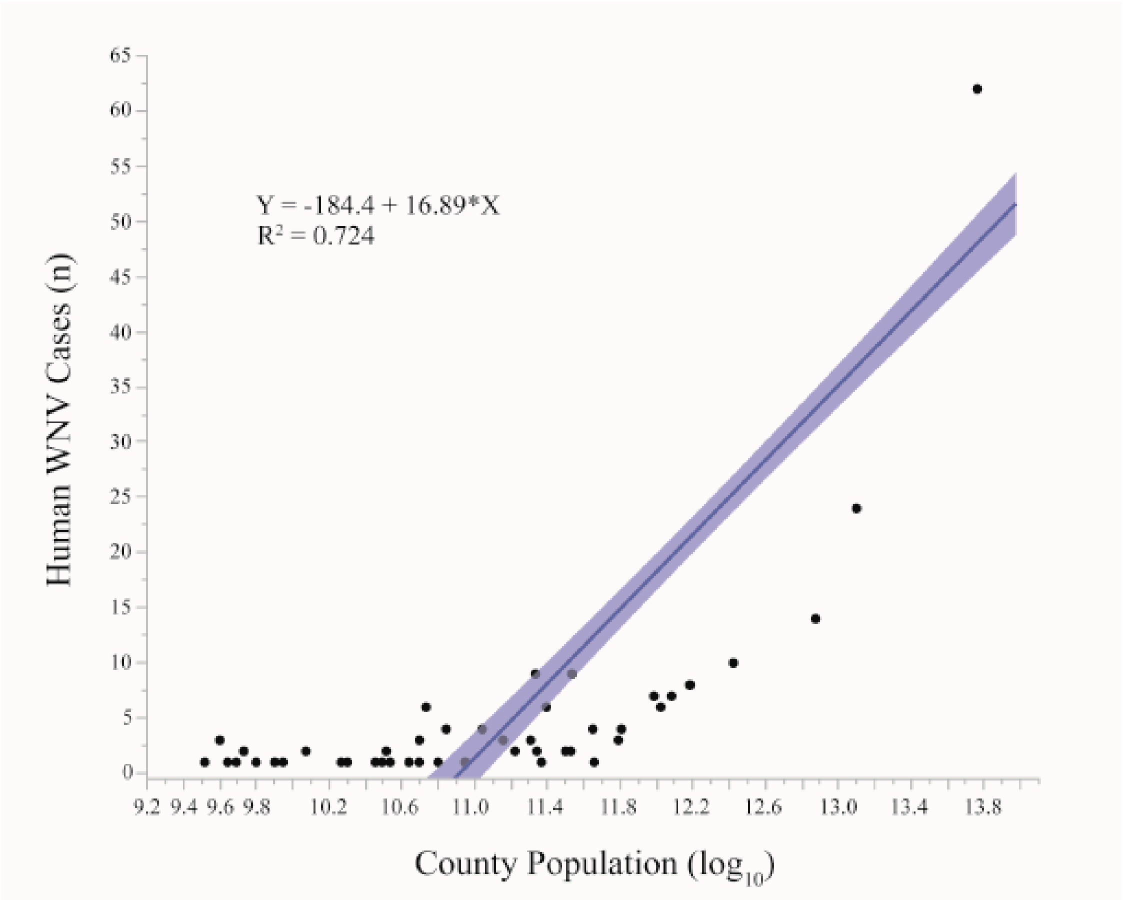
Y = -184.4 + 16.89*X
R² = 0.724
Human WNV Cases (n)
County Population (log10)
0
5
10
15
20
25
30
35
40
45
50
55
60
65
9.2
9.4
9.6
9.8
10.2
10.6
11.0
11.4
11.8
12.2
12.6
13.0
13.4
13.8
